# Mutations altering acetylated residues in the CTD of HIV-1 integrase cause defects in proviral transcription at early times after integration of viral DNA

**DOI:** 10.1371/journal.ppat.1009147

**Published:** 2020-12-22

**Authors:** Shelby Winans, Stephen P. Goff

**Affiliations:** 1 Columbia University, Department of Biochemistry and Molecular Biophysics, New York, New York, United States of America; 2 Columbia University, Department of Microbiology and Immunology, New York, New York, United States of America; 3 Howard Hughes Medical Institute, Columbia University, New York, New York United States of America; Universitätklinikum Heidelberg, GERMANY

## Abstract

The central function of the retroviral integrase protein (IN) is to catalyze the integration of viral DNA into the host genome to form the provirus. The IN protein has also been reported to play a role in a number of other processes throughout the retroviral life cycle such as reverse transcription, nuclear import and particle morphogenesis. Studies have shown that HIV-1 IN is subject to multiple post-translational modifications (PTMs) including acetylation, phosphorylation and SUMOylation. However, the importance of these modifications during infection has been contentious. In this study we attempt to clarify the role of acetylation of HIV-1 IN during the retroviral life cycle. We show that conservative mutation of the known acetylated lysine residues has only a modest effect on reverse transcription and proviral integration efficiency *in vivo*. However, we observe a large defect in successful expression of proviral genes at early times after infection by an acetylation-deficient IN mutant that cannot be explained by delayed integration dynamics. We demonstrate that the difference between the expression of proviruses integrated by an acetylation mutant and WT IN is likely not due to altered integration site distribution but rather directly due to a lower rate of transcription. Further, the effect of the IN mutation on proviral gene expression is independent of the Tat protein or the LTR promoter. At early times after integration when the transcription defect is observed, the LTRs of proviruses integrated by the mutant IN have altered histone modifications as well as reduced IN protein occupancy. Over time as the transcription defect in the mutant virus diminishes, histone modifications on the WT and mutant proviral LTRs reach comparable levels. These results highlight an unexpected role for the IN protein in regulating proviral transcription at early times post-integration.

## Author summary

A key step of the retrovirus life cycle is the insertion of the viral DNA genome into the host cell genome, a process called integration. The process of integration is solely catalyzed by the virally encoded integrase (IN) protein. IN has been reported to influence a number of other viral processes such as reverse transcription, nuclear import and particle morphogenesis. The HIV-1 IN protein is known to be heavily post-translationally modified. In light of the known effect of post-translational modifications on the function of the orthologous proteins of certain retrotransposons, we were motivated to ask how post-translational modifications of HIV-1 IN may regulate its various functions. In this study, we examined the consequences of mutations preventing the acetylation of the IN protein on the retroviral life cycle. Surprisingly, we saw that mutations blocking IN acetylation had only modest effects on viral DNA integration. Instead, we uncovered a novel function for HIV-1 IN in regulating proviral transcription at early times after infection. Our data suggests that IN may be retained on proviral DNA at early times after integration and promote proviral gene expression by altering chromatin modifications at the viral transcriptional promoter.

## Introduction

A key component of a successful retroviral infection is the integration of the proviral genome into the host cell genome. The irreversible process of retroviral integration is imperative for maintenance of infection as it establishes a stable template for proviral transcription and thus continued viral replication. Integration also establishes the provirus as an inextricable component of the host genome, which is a major barrier to treatment and cure of retroviral infections.

The process of integration is catalyzed by the virally encoded integrase (IN) protein. After the viral RNA genome is reverse transcribed to a double-stranded DNA, IN specifically recognizes and binds the ends of the linear viral DNA, consisting of long terminal repeats (LTRs). Integration is a concerted reaction that consists of two catalytic steps–end processing and joining. During end processing, a dinucleotide is cleaved from the 3’ strand at either LTR end exposing a 3’-hydroxyl group that subsequently attacks the host target DNA during the joining process. This leaves a gapped DNA with viral overhangs that must subsequently be repaired by host enzymes [1].

In addition to the main catalytic function of the IN protein, it has long been known that this protein plays additional roles in the viral life cycle [2]. Mutations in IN have been classified into two categories: Class I mutations directly affect the catalytic activity of IN and formation of integrated proviruses, while class II mutations have more pleiotropic effects and perturb late stages of the life cycle including virion maturation [3,4]. Class II IN mutants are defective for viral RNA binding which leads to the formation of eccentric virion particles with mislocalized viral RNA genomes [5,6]. As a consequence of this abnormal particle morphogenesis, virions are defective for early steps of the viral life cycle including reverse transcription and nuclear import [7].

Successful viral integration is important for persistence of the virus, but is also required for efficient transcription of proviral genes [8,9]. Gene expression from unintegrated retroviral DNA is potently silenced via chromatin modification by factors such as the HUSH complex, HDACs, and SETDB1, which act to establish a characteristic silent chromatin composition consisting of high H3K9 tri-methylation and low H3 acetylation [10,11]. This block to transcription is alleviated upon integration into the host genome of permissive cell types [8].

Post-integration transcription of the HIV-1 provirus is regulated by availability of host transcription machinery, and the virally encoded Tat protein, and is reflected in the chromatin state of the viral promoter [12]. HIV-1 proviral transcription can be split into two distinct phases: an early Tat-independent phase and a late, Tat-dependent phase. At early times post-integration, basal transcription generates low levels of viral transcripts encoding viral regulatory proteins including Tat. Once sufficient viral Tat protein has accumulated, it binds the TAR element in the 5’ portion of the viral RNA and through recruitment of factors such as pTEF-B promotes transcription elongation leading to a dramatic increase in proviral transcription [13–15]. By virtue of functioning on a positive regulatory circuit, if transcription initiation is limited, Tat will remain below threshold levels causing a significant reduction in viral gene expression that can lead to entry into a non-productive, latent state [13].

After the HIV-1 DNA is integrated into the host cell genome, it is also subject to the same chromatin-based regulatory mechanisms that modulate host gene expression [16]. Transcription is promoted by histone acetylation at the viral LTR mediated by recruitment of histone acetyltransferases, such as p300 and GCN5, while transcription is suppressed by recruitment of histone deacetylases (HDACs) by various host factors [17–21]. The importance of histone acetylation in regulating viral transcription is evidenced by the successful use of HDAC inhibitors to reactivate latent HIV-1 [22,23]. Further, histone methyltransferases, such as EZH2, deposit the repressive H3K27me3 mark on the LTR of inactive proviruses [24].

Evidence suggests that the site of HIV integration in the human genome plays an additional role in determining proviral transcriptional activity [25]. Retroviral integration in the genome is not a random process. In fact, HIV-1 integration site distribution is significantly biased towards actively transcribed gene regions [26–28]. HIV-1 integration site selection is influenced by binding of the viral pre-integration complex (PIC) to host factors, such as LEDGF, which specifically target integration to active gene regions [29,30]. LEDGF is believed to act largely as a bimodal tether, binding both the PIC and chromatin, thereby recruiting the PIC to chromatin binding sites.

This mechanism of integrase-mediated targeting is seen in other retroviruses as well as retrotransposons [31]. Murine leukemia virus DNAs are targeted to promoter regions of host genes by interactions of IN with Brd2 and 4 [31,32]. Yeast Ty retrotransposons are also targeted precisely to subsets of genomic loci through host factor interactions [33]. For instance, Ty1 and Ty3 elements are both highly targeted to the promoters or transcription start sites of RNA polymerase III transcribed genes by binding of the IN protein to either RNA polymerase III subunits or the Pol III specific transcription factors TFIIIB and TFIIIC respectively [34–36]. Ty5 elements preferentially integrate into heterochromatic regions such as telomeres and the mating type loci [37]. This targeting is mediated by binding of the IN protein to a host heterochromatin protein Sir4 [38]. Interestingly, this binding was found to be contingent upon phosphorylation of the Ty5 IN protein [39]. In the absence of IN phosphorylation, binding affinity for Sir4 decreases and proviral integration targeting is lost, causing the Ty5 retrotransposon to integrate instead throughout the yeast genome.

The HIV-1 IN protein is also known to be post-translationally modified but little evidence to date suggests that these post-translational modifications (PTMs) similarly regulate integration site selection [40]. The best studied of these PTMs is acetylation. IN has been shown to directly interact with histone acetyltransferases, such as p300 and GCN5, which subsequently acetylate four lysine residues in the C-terminal domain (CTD) of IN (K258, K264, K266, K273) [41,42]. Acetylation of the IN protein has been shown to have profound effects on IN strand transfer activity as well as on DNA binding affinity of IN *in vitro* [41]. The effect of blocking IN acetylation *in vivo* has been more contentious. Initial studies reported an extreme defect in integration *in vivo* in the absence of IN acetylation [41]. However, this work made use of a CTD-tagged IN protein. Considering the proximity of the tag to the locations of acetylation (K264, K266, K273), these data were called into question when a second group reported only very mild integration defects in the absence of acetylation of an untagged IN protein [43].

In this study, we conservatively mutate the four known acetylated lysine residues in the CTD of HIV-1 IN. We show that ablating acetylation of the HIV-1 IN protein has only modest effects on early steps of the retroviral life cycle such as reverse transcription and integration. However, we observe a severe defect in transcription of proviral genes at early times after infection by a virus expressing an acetylation-deficient IN mutant. The observed transcriptional defect present in proviruses integrated by an acetylation-deficient mutant IN cannot be explained by delayed integration dynamics in the mutant and is also not confined to the context of Tat-dependent transcription or even the LTR promoter. Mapping the genomic locations of proviruses integrated by either the acetylation mutant or WT IN reveals that these differences are not due to altered integration site selection but rather to a lower rate of transcription. The poor transcription is accompanied by the presence of altered histone modifications present on the viral LTR. We observe significantly less of the active chromatin mark of H3 acetylation on the LTRs of proviruses formed by an acetylation-deficient IN. The transcription defect in the acetylation-deficient mutant virus diminishes over time and the presence of active chromatin associated histone modifications correspondingly returns to wild-type levels. Further, these mutations in IN decrease the occupancy of the IN protein on the proviral DNA suggesting that IN may normally be retained on the viral DNA after integration and plays an active role in establishing a permissive chromatin environment for transcription. To our knowledge this is the first evidence that the IN protein can influence proviral transcription post-integration and raises the possibility that PTMs of IN, such as acetylation, may coordinate this function.

## Results

### Mutations of acetylated IN residues affect HIV-1-mediated transduction

HIV-1 integrase has four lysine residues in the C-terminal domain known to be acetylated–K258, K264, K266 and K273 [41,42]. We conservatively mutated each lysine residue to arginine either individually, or in combination to form the quadruple acetylation (QA) mutant in the pNL4.3R-E- luciferase reporter viral vector. We then prepared mutant or WT virus pseudotyped by VSV-G envelope by co-transfecting HEK293T cells with plasmids encoding the HIV-1 genomes and the VSV-G envelope. There were no significant changes in the yields of virus for any of the mutants relative to the WT control as judged by viral RNA genome content.

**Fig 1:**
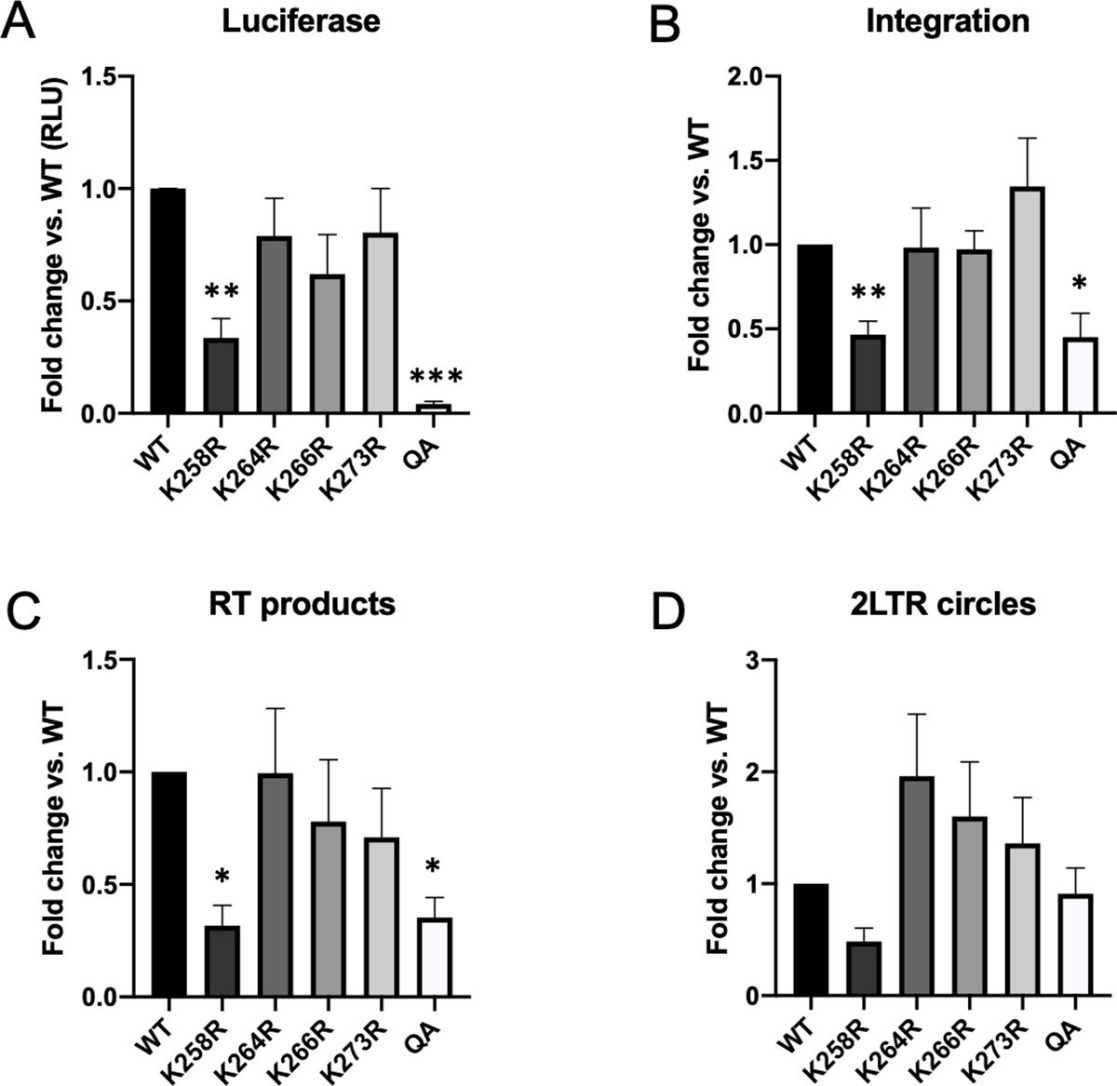
Blocking acetylation of HIV-1 IN affects successful transduction of HeLa cells while causing only modest defects in early viral replication steps. Acetylation-site mutations were introduced into a pNL4.3R-E- luciferase reporter genome and used to prepare virus. HeLa cells were infected with fresh viral supernatant and collected at 48 hours post-infection. (A) Acetylation deficient mutant IN (QA) virus had major defect in successful viral transduction as measured by luciferase reporter readout. The K258R point mutation caused a significant 3-fold reduction in overall viral transduction. Other point mutations introduced in this region had no effect. Viral DNA intermediates were subsequently assayed using qPCR-based approaches. (B) Integrated provirus was quantified using an Alu-gag nested PCR approach and normalized to a housekeeping gene (GAPDH). Viruses carrying the K258R or QA mutant IN had a modest 2–3 fold defect in integration. (C) Products of reverse transcription (RT) were assayed by qPCR using LTR specific primers and normalized to GAPDH. Viruses carrying the K258R or QA mutant IN demonstrated an approximate 3-fold defect in reverse transcription. (D) 2-LTR circles, a dead end viral product that marks nuclear entry, were quantified using primers that span the LTR-LTR junction. No significant differences between WT and acetylation defective mutant viruses were observed. Data shown is average +/- SEs of a minimum of five independent experiments run in duplicate. Statistical significance was measured using a one-way ANOVA with Bonferroni correction for multiple comparisons, followed by a pairwise Welch’s t-test assuming unequal variance (* p < 0.01, **p<0.001, ***p<0.0001).

HeLa cells were infected with equal quantities of fresh virus and successful transduction was quantified two days post-infection using a luciferase reporter assay (Fig 1A). Mutation of single lysine residues had little effect on viral transduction relative to the WT control. The K258R point mutation in IN did cause a significant 3-fold reduction in luciferase reporter signal. However, viruses expressing the combined quadruple acetylation (QA) mutant IN had a significantly larger defect with an average 50-fold decrease in luciferase signal (p < 0.0001).

### Viruses carrying an acetylation deficient mutant IN (QA) have only modest defects in early viral replication steps

Previous reports, while controversial, also described reductions in reporter gene expression from acetylation-deficient IN mutants, and attributed this difference to integration defects [41]. To determine if the reduction in luciferase signal observed in the QA mutant viral infection was due to a decrease in DNA integration efficiency, we harvested cellular DNA from infected HeLa cells at two days post-infection and quantified integration frequency using an Alu-gag qPCR approach [44]. We found that the acetylation deficient QA IN mutation led to only a modest (2-fold average) decrease in integration efficiency relative to the wild-type control (Fig 1B). This decrease did not account for the 50-fold decrease in expression. All but one of the individual point mutations in IN caused no detectable defect in integration as compared to WT.

Residues in the CTD of IN have also previously been identified as class II mutants that are defective for viral RNA binding and particle morphogenesis, and as a consequence the virions are defective in early steps of infection such as reverse transcription and nuclear import [7]. To determine if our mutations might display a similar phenotype we assayed the early steps of the viral life cycle. We quantified both the total reverse transcription (RT) products and 2-LTR circular DNAs, a dead-end viral DNA product formed upon nuclear entry (Fig 1C and 1D). There was a small, but reproducible 3-fold decrease in the abundance of total RT products in the K258R and QA mutants. This decrease in RT products likely accounts for the decrease seen in the levels of integrated DNA. If the Alu-gag qPCR signal is normalized to reverse transcribed viral DNA available for integration, the acetylation deficient IN mutants display no defect at all as compared to WT, suggesting that the mutant IN retains near wild type levels of catalytic activity. Further, all mutants had a comparable level of 2-LTR circles, indicating that nuclear import was not affected by the IN mutations. When the catalytic activity of IN is impaired by class I mutations, 2-LTR circles are known to accumulate in the abortively infected cells. Thus, the finding here of comparable levels of 2-LTR circles in cells infected with viruses carrying the WT and QA mutant IN proteins supports the notion that the catalytic activity of the acetylation deficient mutant IN is not severely impaired.

### Transcription from proviruses formed by the acetylation-deficient QA mutant IN is significantly reduced

**Fig 2:**
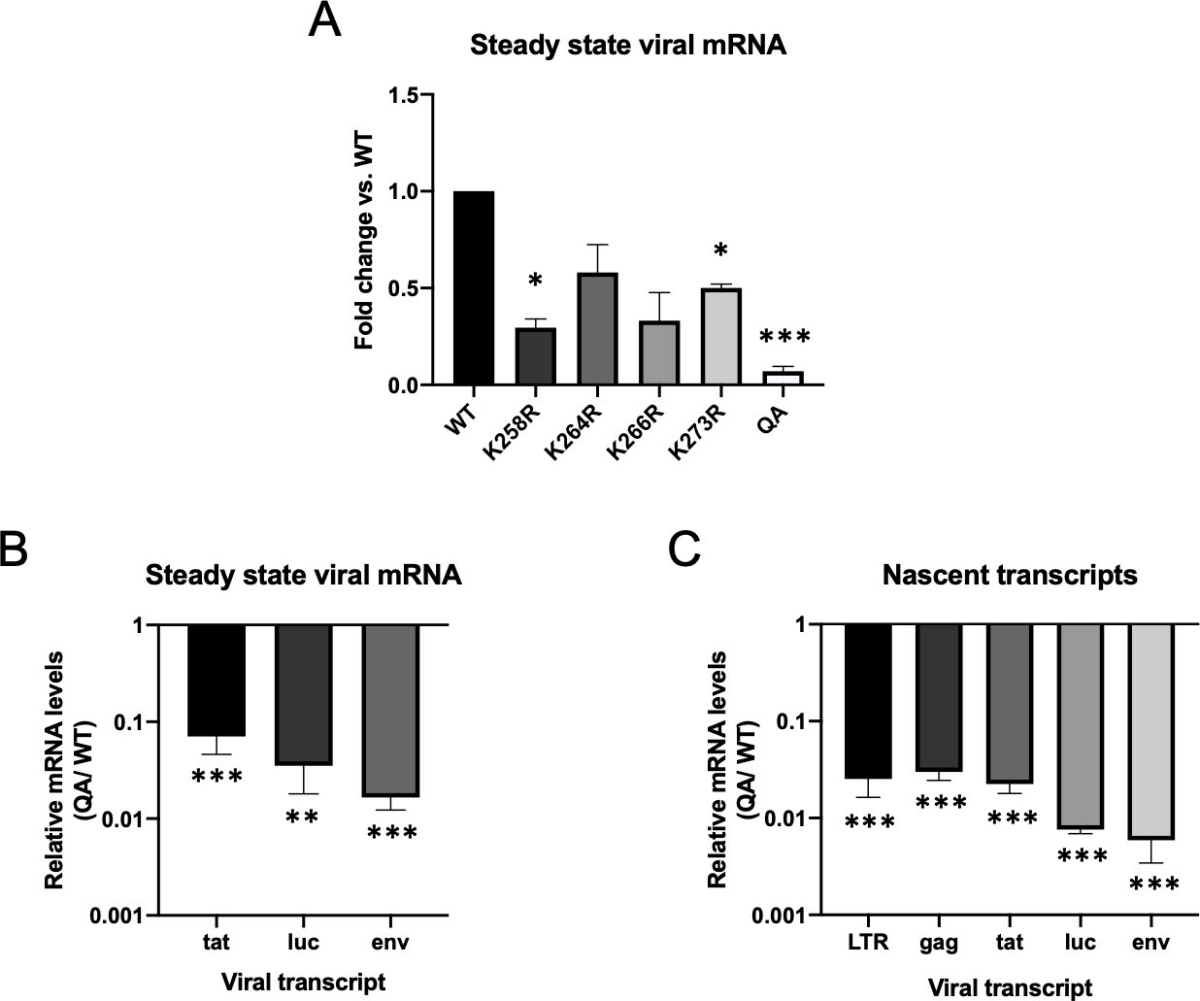
Proviruses integrated by an acetylation-deficient IN mutant (QA) exhibit significant reduction in viral gene transcription. (A) Steady-state viral mRNA levels were measured by qRT-PCR on total cellular RNA collected from infected HeLa cells at two days post-infection. Data shown are levels of viral mRNA as measured by primers amplifying spliced *tat* message. Detected viral RNA levels were normalized to viral DNA content to adjust for discrepancies in viral DNA available for transcription. Viral mRNA levels were significantly reduced from proviruses integrated by an acetylation deficient mutant IN (QA) (50 fold). Individual point mutations in IN had a more modest defect in viral mRNA levels ranging from 2-4-fold on average. Data shown are averages +/- SEs of three independent experiments run in duplicate. Statistical significance was assayed using a one-way ANOVA analysis. (B) Steady state viral mRNA levels produced from provirus integrated by the QA mutant IN vs. WT IN were measured using primers against various other viral transcripts and normalized to total viral DNA. Data is shown as a ratio of RNA levels detected from QA IN mutant virus vs. WT (C) Rate of transcription was measured using 5-ethynyl uridine (EU)-labeling of nascent transcripts followed by mRNA quantification using RT-qPCR with primers against various viral transcripts normalized to a housekeeping gene (GAPDH). Shown is the ratio in transcripts detected from proviruses integrated by a QA mutant IN vs. WT in a minimum of three independent experiments run in duplicate. All viral transcripts were reduced from proviruses integrated by the acetylation deficient mutant IN at high significance (80-fold average). Significance was measured using a paired t-test (* p < 0.01, **p<0.001, ***p<0.0001).

Though the early steps of the retroviral life cycle, up to and including integration, were only modestly affected by the acetylation deficient IN mutant (QA), the luciferase reporter assays suggested a large defect in successful retroviral transduction. We therefore tested the effect of the QA mutation on the next step of the viral life cycle–proviral transcription. We measured steady state mRNA levels of various viral genes at 48 hours after infection in HeLa cells (Fig 2A, *tat* shown). To account for any differences in levels of integrated proviral DNA, the viral mRNA levels were normalized to viral DNA content as measured by qPCR. We observed a highly significant decrease in global viral mRNA levels from proviruses formed by the acetylation deficient QA mutant IN (20-fold, p<0.0001). To ensure that our introduced mutations were not uniquely affecting *tat* message, we also quantified steady state levels of other viral transcripts (Fig 2B). Both envelope and luciferase mRNA levels were similarly reduced.

To determine if the observed difference in steady state mRNA levels was due to differences in the rate of transcription and not post-transcriptional effects such as differential RNA decay, we performed a 5-ethynyl-uridine (EU) pulse-labeling experiment. This allows us to more directly measure differences in newly synthesized proviral transcripts. Cells were infected with QA mutant and WT virus preparations, and at 48 hours post-infection were incubated for 4 hours with EU to label nascent transcripts. Total cellular RNA was harvested and labeled RNA was subsequently isolated. Viral-specific RNA was quantified by qRT-PCR using primers against various transcripts and normalized to total viral DNA. We observed a substantial 80-fold average decrease in nascent viral transcripts produced from proviruses integrated by QA mutant IN vs. WT IN regardless of the viral message quantified (Fig 2C, p<0.0001).

These experiments show that the QA mutation that blocks known acetylation of IN has little effect on early viral replication steps but severely impairs proviral transcription. To ensure that this phenotype is not limited to the context of HeLa cells in which the majority of the experiments shown here have been performed, we also infected HEK293T, CEM and Jurkat cells. From these infections, we quantified luciferase activity, reverse transcription and integration efficiency as well as transcription in parallel (S1 Fig). We see similar phenotypes, albeit to differing extents, in all of these lines indicating that this is not a cell-type or cell-line specific phenomenon.

### The transcription defect induced by the QA mutation in IN is limited to early times post-infection

**Fig 3:**
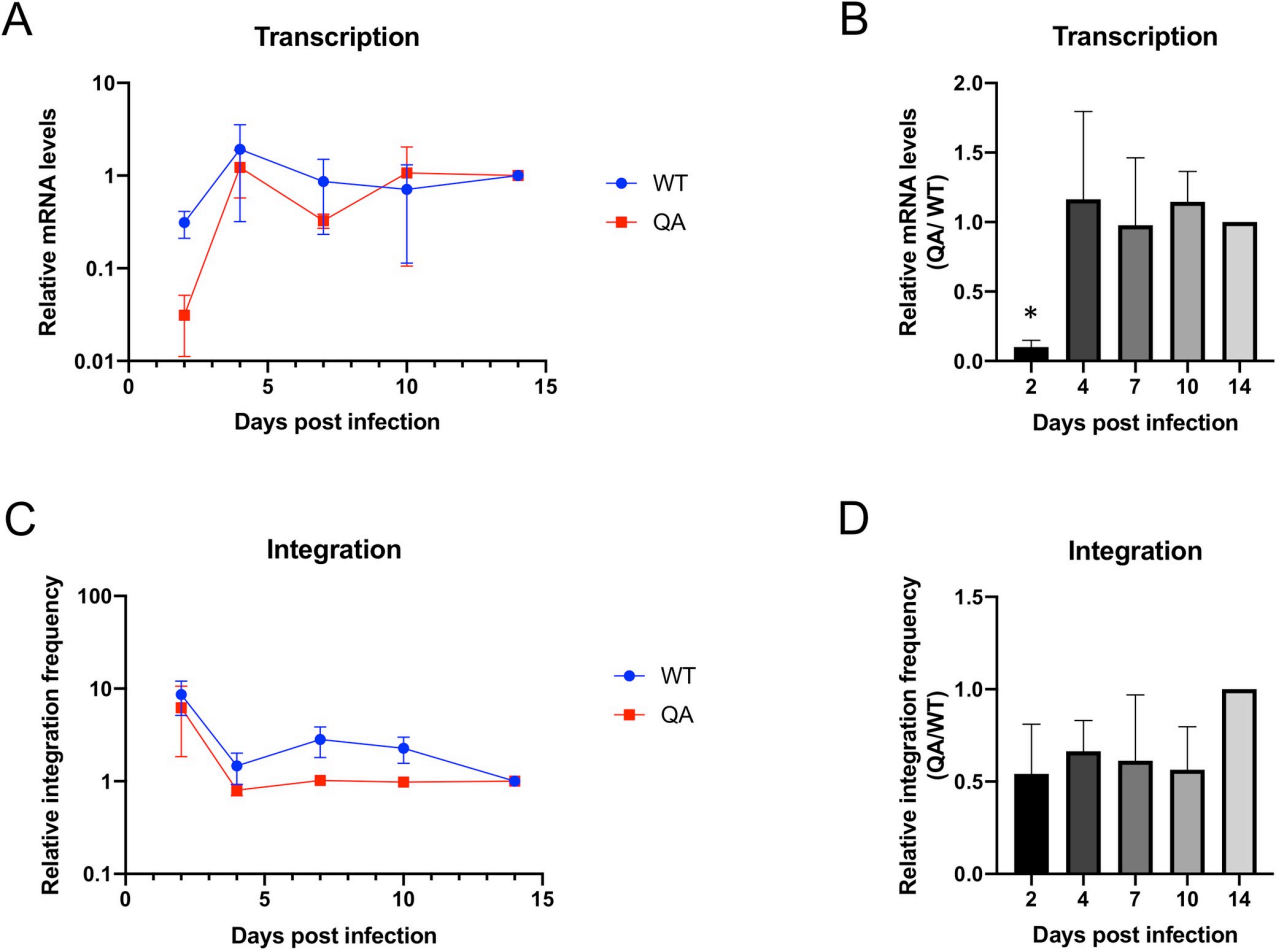
Transcription from proviruses integrated by QA mutant IN is delayed relative to WT despite comparable integration timing. HeLa cells were infected with WT or QA mutant virus and DNA and RNA were collected at various time points ranging from 2 to 14 days. (A) Steady state viral mRNA levels were measured over the time course using qRT-PCR with primers targeting spliced *tat* message. Transcript levels were normalized to total viral DNA levels and the fold change in viral mRNA levels over time relative to the final time point (14 dpi) was quantified for WT and mutant viruses individually using the 2^-ΔΔCt^ method and normalized to total viral DNA. Values are plotted on log scale to highlight the low value of the QA sample at early times. (B) Same data expressed as a ratio of detectable mRNA levels in cells infected by the QA mutant virus vs. WT virus at each time point. (C) Integrated proviral DNA was measured and quantified at the same time points using an Alu-gag based qPCR. The fold change in integrated provirus levels over time relative to the final time point (14 dpi) was quantified using the 2^-ΔΔCt^ method and normalized to a housekeeping gene. (D) Same data expressed as a ratio of detectable proviral integrants in cells infected by the QA mutant virus vs. WT virus at each time point. Shown is the average +/- SEs from three independent biological replicates performed in duplicate. Statistical analysis of time course samples was assayed using a paired t-test comparing WT to QA mutant virus at individual time points (* p < 0.01).

To test whether the decrease in expression of proviruses integrated by the QA mutant IN was long-lived, we assessed proviral transcription by measuring *tat* mRNA levels at increasing times post-infection by the QA mutant and WT virus. To avoid overstating any defective phenotype, we chose to monitor the spliced *tat* mRNA, which was amongst the least reduced viral transcript measured in previous experiments (Fig 2B and 2C). We observed that viral mRNA levels were severely reduced in the mutant as compared to WT virus at two days post-infection, but began to increase at later time points (Fig 3A and 3B). By four days post-infection, mRNA levels produced from the QA mutant provirus rose to that of WT and remained at this level until at least 14 days post-infection. Thus, the defect in proviral transcription of the mutant virus is limited to early time points after infection.

It is possible that viral replication is merely delayed in viruses carrying the acetylation deficient mutant IN and thus an increase in viral transcription at later time points may be a reflection of delayed completion of integration. To determine if viral integration dynamics varied between viruses carrying WT or acetylation deficient (QA) mutant IN we monitored detectable proviral integration over a 14-day time course using an Alu-gag nested qPCR approach (Fig 3C and 3D). Consistent with initial experiments, we observe an approximate 2-fold reduction in detectable integrated proviruses at two days post-infection (Fig 3C and 3D). In cells transduced with mutant virus, we consistently observe about a 2-fold decrease in integration frequency over the entire time course indicating that the mutation does not cause a delay in integration. This suggests that the altered transcription dynamics displayed by viruses with mutant IN are not due to differences in integration timing.

### Defect in proviral transcription of acetylation-deficient IN mutant is independent of the LTR promoter

The pNL4.3 viral vector utilized to read out the effects of IN mutations on the level of transduction generates virion particles with mutant IN protein, and thus monitors the function of that protein during the early steps of infection. But in addition, the provirus formed by infection carries the same mutations, and thus changes in the level of reporter gene expression, in principle, could be mediated by effects on viral mRNA processing or regulation. This is not implausible, because many of the IN mutations are located in regions of the viral genome utilized for splicing, generating Tat and Rev proteins, and HIV-1 proviral transcription relies heavily on the presence of sufficient Tat protein [45]. If mutations in the IN open reading frame perturb *tat* mRNA splicing or expression, then a deficit of Tat protein would cause a dramatic decrease in proviral transcription regardless of the mutation status of the IN protein.

To determine if the effect of the acetylation-deficient mutant IN on transcription is genuinely due to the mutant IN protein or to defects in splicing dynamics, we made use of a reporter gene driven by a Tat-independent, CMV promoter. Virus was made by co-expressing a plasmid expressing HIV-1 Gag-Pol protein with either the WT or QA mutant IN sequences, along with a minimal transducing viral genome carrying a CMV-driven ZsGreen reporter gene. This allows us to assay reporter gene expression from a provirus that is integrated by WT or QA mutant IN but whose expression is independent of Tat protein levels and even independent of the proviral LTR promoter. Further, the mutant IN will only be present in the form of the incoming protein, as the reporter encodes no *gag-pol* allowing us to separate out the mutation from the integrated viral DNA sequence.

**Fig 4:**
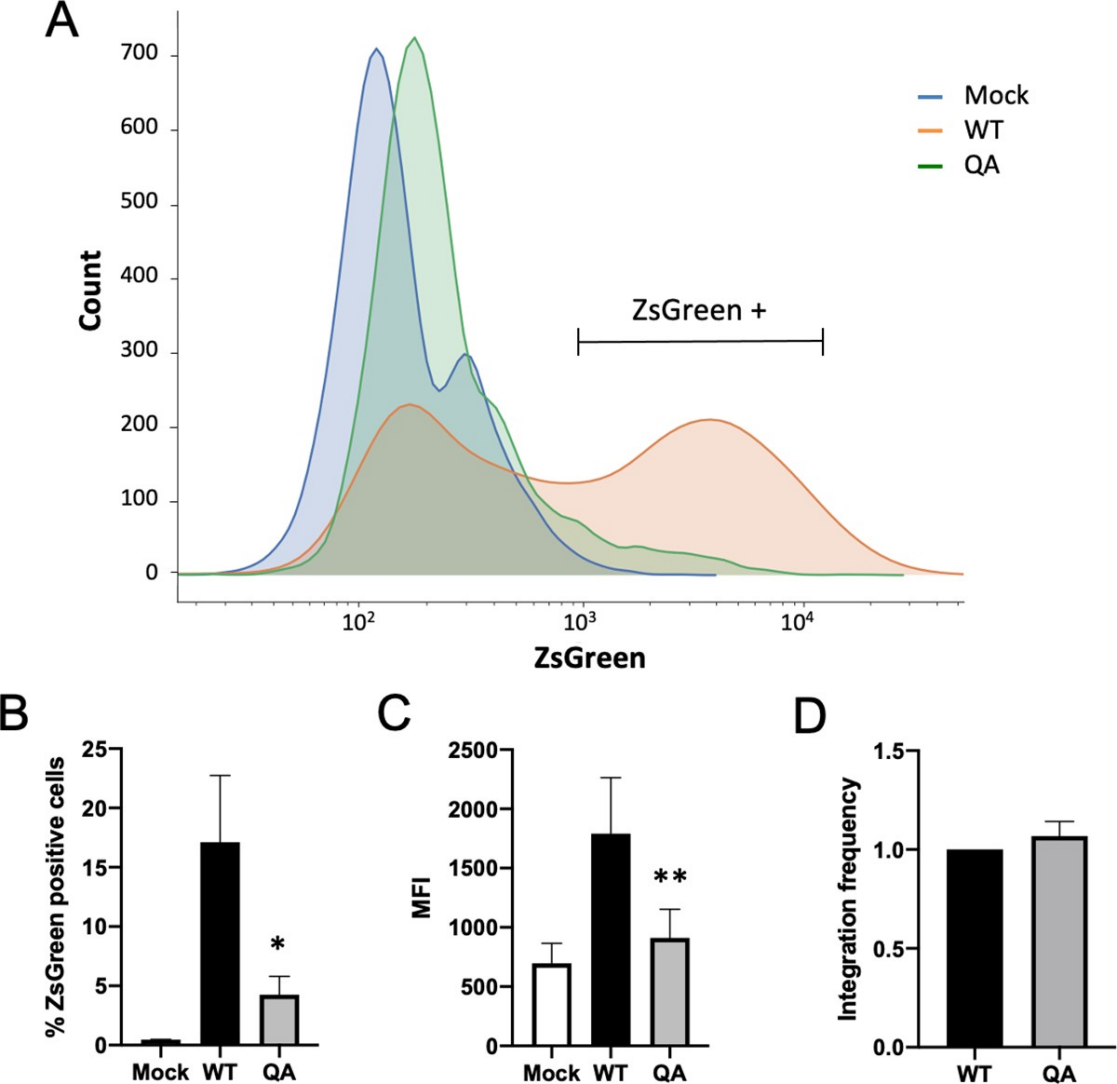
The effect of acetylation-deficient mutant IN on proviral transcription is independent of Tat and the LTR promoter. The QA mutation in the CTD of IN was introduced into pCMV-delta-R8.9 vector expressing only HIV-1 *gag-pol*. VSV-G psuedotyped virions were produced by co-transfecting the plasmid expressing WT or QA mutant *gag-pol* along with a minimal transducing ZsGreen reporter driven by a CMV promoter and a plasmid expressing the VSV-G envelope. HeLa cells were infected with these preparations and samples were collected two days post-infection. (A) ZsGreen expression profiles of infected cells. Representative flow cytometry data of one independent experiment is shown. (B) Average percent of ZsGreen positive cells and (C) mean fluorescence intensity (MFI) after infection with viruses carrying WT or QA mutant IN. Data shown are averages +/- SEs of four independent experiments run in duplicate. Statistical significance was gauged using a paired t-test comparing WT to QA mutant virus at individual time points (* p < 0.05, **p<0.01).

At 2 days post-infection, we performed flow cytometry on infected HeLa cells to quantify ZsGreen expression. The expression from the proviral ZsGreen reporter gene integrated by the acetylation-deficient QA mutant IN was dramatically decreased relative to proviruses integrated by WT IN. In cells infected with virus possessing a QA mutant IN, there were substantially fewer ZsGreen positive cells (Fig 4A and 4B, 10-fold), and of the cells that are positive, the mean fluorescence intensity (MFI) was also decreased (Fig 4C) despite viral integration being comparable (Fig 4D).

The fact that integration by the QA mutant IN still induces a transcriptional defect of this CMV-driven ZsGreen reporter strongly suggests that the observed phenotype is not a product of perturbation to Tat levels. However, the integrated minimal viral genome in this experiment still contains LTR sequences that could somehow mediate this phenotype. To further remove any effect of the LTR promoter, we repeated this experiment using a self inactivating (SIN) viral genome carrying a GFP reporter gene expressed from a human PGK promoter. Unlike the previous CMV-driven reporter, the integrated SIN-GFP proviral genome has no functional LTR. Even in this context we still observe a strong defect in viral gene expression at two days post-infection despite comparable levels of integrated DNA (S2 Fig).

These data demonstrate that the large effects of the introduced QA IN mutation on proviral expression are attributable to the mutant IN protein itself and are not Tat-dependent or confined to the context of the LTR promoter. These experiments exclude the possibility that the observed transcriptional defect is a byproduct of perturbations of viral regulatory mRNA processing or changes in the levels of Tat protein. The effect is seen with multiple vectors utilizing varying promoters, and in multiple cell lines.

### Proviruses formed by a QA mutant IN have similar integration distribution to that of WT proviruses

**Fig 5:**
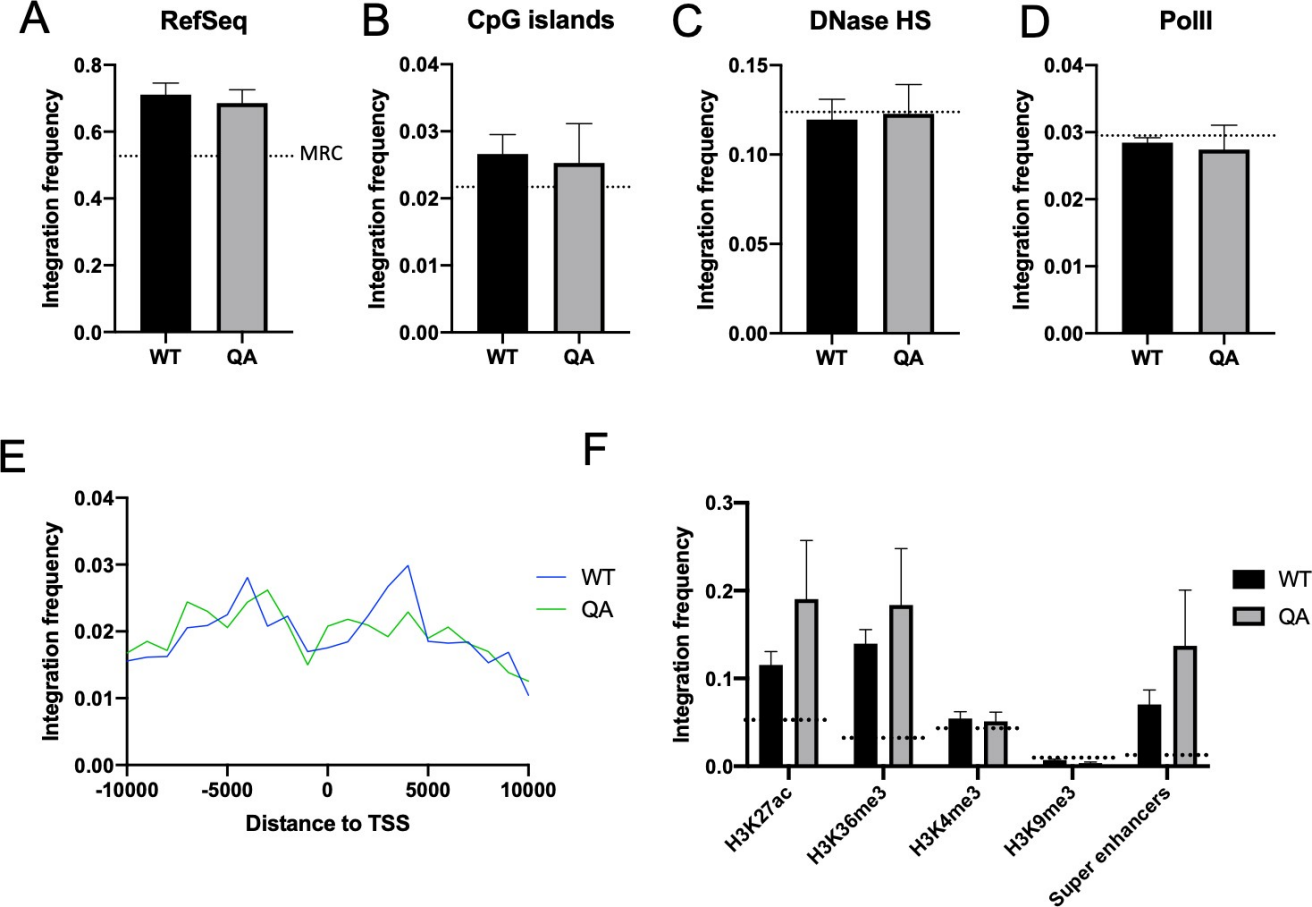
Integration site distribution of proviruses integrated by either an acetylation deficient (QA) mutant or WT IN is similar. HeLa cells were infected with pNL4.3R-E- virus carrying either WT IN or the QA mutant IN and collected at two days post-infection. Genomic DNA was isolated from three independent biological replicates and used to construct next generation sequencing (NGS) libraries to map integration sites. Frequency of integration sites falling within annotated RefSeq genes (A), or within 1 kb of annotated CpG islands (B), or DNase hypersensitivity sites (C) was calculated using BedWindow. (D) Integration sites were compared to RNA polymerase II binding sites as determined from ENCODE ChIP-seq data. (E) Distance of each integration site to the nearest annotated transcription start site (TSS) was determined using BedClosest tool from BedTools suite. A 10 kb window around the TSS is shown. (F) Genomic coordinates of common histone modifications in HeLa cells were determined from ENCODE ChIP-seq data. Coordinates of “super enhancers” in HeLa cells were extracted from the SEA 3.0 database [71]. Frequency of integration within 1 kb of these locations was calculated. Frequency of integrations detected in a matched random control (MRC) data set for each analysis is shown as a dashed line. Data shown is average +/- SEs of three independent experiments. Statistical significance was assayed using a paired t-test (S2 Table). No significant differences in integration site distribution were detected between WT and mutant QA proviruses.

The data presented thus far indicate that proviruses integrated by an acetylation-deficient mutant IN have a significantly lower rate of transcription. This effect is not due to decreased frequency of integration and is independent of the Tat protein or the viral LTR promoter. If the mutations ablating IN acetylation are not affecting IN catalytic activity, another plausible explanation is that these mutations may be affecting integration site targeting. If acetylation deficient IN alters integration site selection, then transcription from these mutant proviruses could be reduced compared to WT proviruses due to chromosomal position effects. WT IN normally binds LEDGF and targets integration to active gene regions [30]. The mutant IN might bind host factors, such as LEDGF, differently than WT IN and thus might have altered integration site preferences that affect subsequent transcription of the integrated proviruses. To determine if this is the case, we mapped integration site distribution in cells infected with WT or QA mutant virus using a high-throughput sequencing approach (Fig 5, S1 Table).

We collected genomic DNA from independent infections in HeLa cells and amplified the viral–host genome junctions using nested rounds of PCR. Subsequent high throughput sequencing of the viral-host junctions allowed us to map viral integration sites in the human genome and to compare locations of integrations with various genomic features such as annotated genes, transcription start sites and CpG islands as well as the pre-infection genomic locations of common proteins or histone modifications as deduced from ChIP-seq data. Three distinct NGS runs were performed from independent infections to account for biological variation. Integration frequency near features was determined for each run and is presented as an average of the repeat experiments. Differences in integration site distribution of proviruses formed by WT or QA IN were gauged for significance relative to each other as well as relative to a matched random control (MRC) using a paired t-test (S2 Table).

We observed that the WT and QA mutant proviruses have a similar distribution of integration sites. Both mutant and WT proviruses integrated with equivalent frequencies near RefSeq genes, CpG islands, DNase hypersensitive regions and RNA polymerase II binding sites (Fig 5A-5D). The distribution of integration sites around known transcription start sites (TSS) was also comparable between the WT and mutant proviruses (Fig 5E). We correlated viral integration sites with different chromatin modifications that mark either active or repressive chromatin environments (Fig 5F). We saw only subtle differences in integration preferences, particularly with respect to overlap with the known locations of pre-infection active chromatin marks such as H3K27ac and H3K36me3, and neither of these differences rose to the level of statistical significance. Recent work has shown that HIV-1 integrates frequently near super-enhancers in T-cells [46]. Super-enhancers are marked by high levels of H3K27ac as well as transcriptional activators such as Brd4 and the histone acetyltransferase p300 [46,47]. To determine if our acetylation deficient IN mutant affected integration near super-enhancers, we quantified the number of integrations detected in the immediate proximity of known super-enhancers and found no significant difference in the frequency of integration in these regions between the WT and mutant IN. Proviruses integrated by the QA mutant IN actually seem to have a slight preference for integration near these active chromatin marks as well as super-enhancers which does not explain the profound defect observed in proviral transcription. Thus, we conclude that the substantial reduction in proviral expression induced by the QA IN mutation is not likely explained by changes in the integration site profile of the provirus.

### QA mutant proviral DNA is associated with less IN and lower levels of active chromatin marks

**Fig 6:**
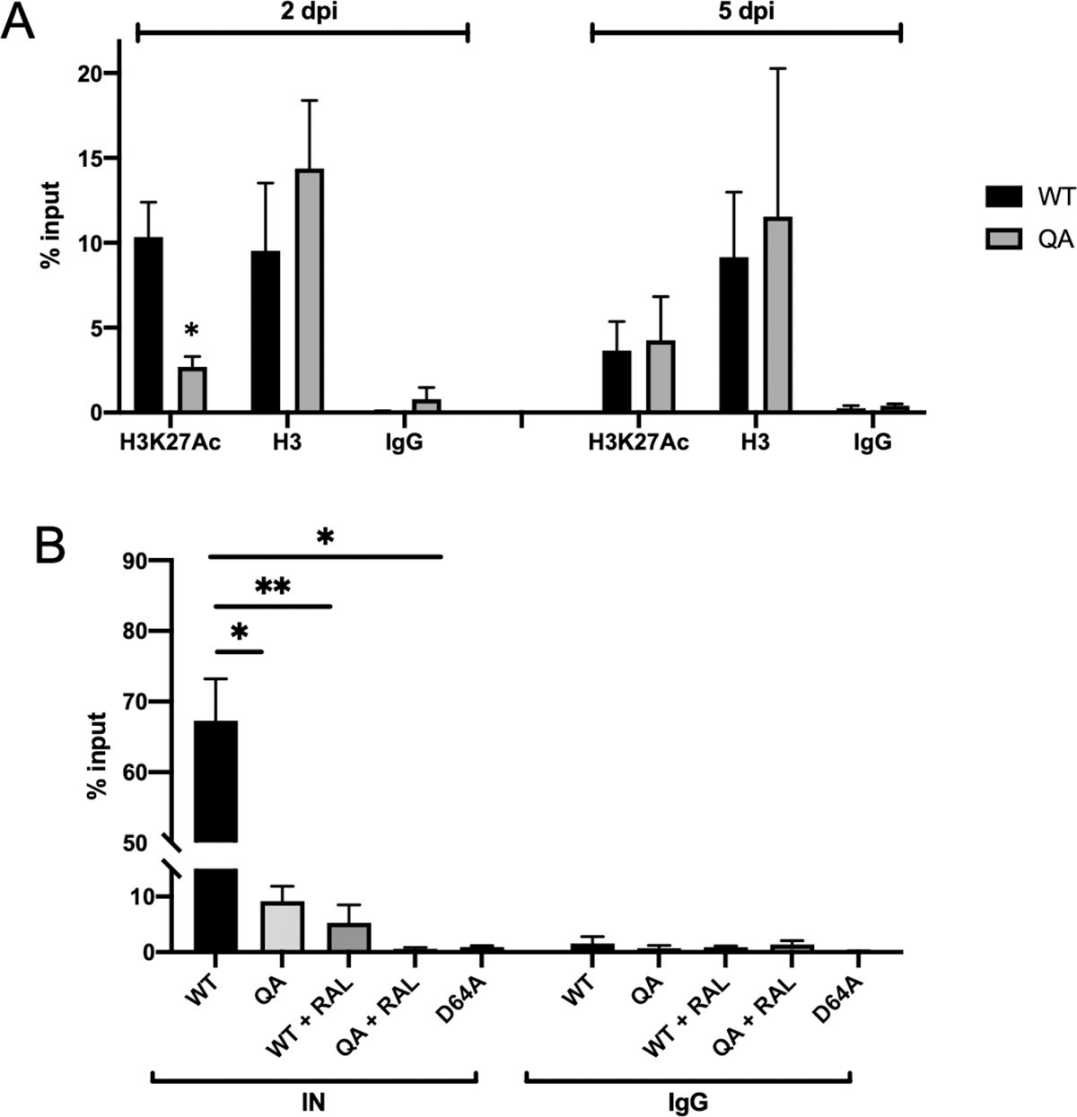
Proviruses integrated by QA mutant IN are less associated with an active chromatin mark, H3K27ac and with IN protein, than WT viruses. Viral DNAs associated with the active histone mark H3K27ac, with total histone H3, or with IN protein were quantified from infected HeLa cells via chromatin immunoprecipitation (ChIP) followed by qPCR using LTR specific primers. Data are shown as a percent relative to input DNA levels. (A) At two days post-infection, there was significantly less DNA of proviruses integrated by the QA mutant IN bound to H3K27ac as compared to WT control. In samples collected at five days post-infection from the same initial infections, quantities of viral DNA bound to H3K27ac for proviruses integrated by both WT and QA mutant IN reached comparable levels. Levels of viral DNAs associated with total histone H3 was comparable on mutant and WT LTRs at both time points. Data shown are the averages +/- SEs of three independent experiments run in duplicate. Statistical significance was gauged by unpaired t-test (* p < 0.01). (B) At 24 hours post-infection, significantly less viral DNA of the QA mutant was immunoprecipitated with antibody to IN. Raltegravir treatment during both WT and mutant virus infection resulted in significantly less viral DNA bound to IN at 24 hours post-infection. Similarly, a class I catalytic IN mutation (D64A) also results in less IN occupancy at the viral LTR. Statistical significance was gauged by unpaired t-test (* p < 0.01, **p<0.001). Results shown are averages +/- SEs of two independent experiments run in duplicate.

Mapping of integration sites in infected cells allowed us to determine preferences for integration near the location of specific pre-infection histone modifications but does not report the histone modifications on the viral LTR itself. To look directly at the histone modifications present on the viral DNA we performed chromatin immunoprecipitation with antibodies specific for total or acetylated histone H3 followed by qPCR with LTR specific primers. At early points after infection (2 dpi), the WT proviral DNA was heavily loaded with acetylated histone H3 (H3K27ac), a chromatin mark known to be associated with active chromatin. Significantly less of the QA mutant proviral LTR than the WT LTR was associated with acetylated histone H3 (Fig 6A, p<0.05). The effect of the mutant IN on chromatin modifications present at the viral LTR was transient, and by five days post-infection, the levels of the mutant LTR with H3K27ac were comparable to WT (Fig 6A). Thus, the acetylation deficient mutant IN protein established proviruses that were poorly marked for active transcription immediately after infection, but over time the proviruses acquired normal histone modifications and high level expression. As a further control, we observed that comparable amounts of mutant and WT proviral DNAs were associated with total histone H3 demonstrating that there was no defect in the loading of histones onto the viral DNA.

The IN protein could affect the local chromatin environment on the viral LTR in a number of ways. First, IN is known to interact with and recruit many host cell factors that aid in the process of integration and it seems plausible that the introduced mutation may disrupt IN-host factor interactions that could lead to changes in chromatin. Second, the QA mutation in IN may affect binding or retention of the IN protein on the viral DNA itself after integration and thereby fail to recruit necessary machinery for active transcription.

To differentiate between these possibilities we first immunoprecipitated either WT or QA mutant IN proteins and performed a mass-spectrometry screen of the associated proteins to identify differentially binding host factors (data not shown). The repertoire of host factors that precipitated with both IN proteins was nearly identical, with no single host factor detected with significantly differential binding. The result made it unlikely that differential host factor interaction could cause such a large discrepancy in proviral expression levels.

Recently, it has been shown that the PFV intasome is stabilized on the provirus after strand transfer [48]. It is not currently known whether the HIV-1 IN protein remains bound to the provirus after integration nor whether it might affect the state of chromatinization or the subsequent binding of transcription machinery to the provirus. *In vitro* studies on acetylation-deficient IN have shown that acetylation of IN promotes viral DNA binding [41]. Thus, while our acetylation deficient QA mutant IN is still catalytically active, it is possible that the protein is not retained on the viral DNA after catalysis as the WT protein might be, and thus might fail to properly promote expression.

To determine if WT IN is in fact bound to the provirus, and if ablating acetylation of IN affected the binding to viral DNA *in vivo*, we infected HeLa cells with mutant and WT virus preparations, and performed chromatin immunoprecipitation (ChIP) of genomic DNA using antibodies against IN, and scored for the recovery of viral DNA sequences by qPCR. We found that the IN protein was indeed associated with viral DNA in these assays. Importantly, we detected significantly less viral DNA bound by the acetylation-deficient QA IN as compared to WT (Fig 6B). While the IN antibody used for ChIP was validated for comparable binding to both WT and mutant IN via Western blot, we wanted to ensure that the differential precipitation observed with the WT and mutant IN proteins was not simply due to differential binding of the monoclonal antibody to the proteins themselves. Thus, we repeated the ChIP protocol on multiple unique biological replicates with a polyclonal antibody against IN (S3 Fig). Use of the polyclonal antibody demonstrated the same phenomena, indicating that retention of the QA mutant IN protein on the viral DNA is less than that of the WT protein.

We hypothesize that the WT IN protein is retained on the viral DNA after integration of the provirus, and acts to promote early viral transcription by influencing chromatin modifications at the viral LTR promoter. When we introduce the QA mutation, the binding of IN to the viral DNA is perturbed, and thus it is not retained as well and fails to promote transcription leading to the observed early expression defect in our mutant virus. However, it is difficult to distinguish whether the IN binding measured by ChIP is on integrated or unintegrated viral DNA. Due to the inherent efficiencies of ChIP, it is not possible to first IP the IN protein and then perform traditional Alu-gag PCR on the recovered DNA. We could, however, assay IN retention on exclusively unintegrated DNA. We performed IN ChIP on cells infected in the presence of raltegravir to block integration, as well as in cells infected with the class I catalytic IN mutant D64A, and compared the levels of viral DNA bound to IN after WT infection (Fig 6B). We observed that in this setting there was less DNA bound by the QA mutant IN than by the WT IN. Thus, although the QA mutant was able to catalyze integration normally, it was more rapidly lost from any unintegrated DNA.

We also noted that there was significantly less IN bound to the unintegrated DNA in both raltegravir treated samples and D64A infected cells than to the integrated DNA, for both QA and WT IN. This suggests that integrase is lost from the unintegrated DNA when integration is blocked, and that more integrase is actually retained on the integrated proviral DNA. The result also indicates that much of the viral DNA bound to IN in our ChIP assay in WT infection is in the context of the integrated provirus. In sum, the findings support the hypothesis that the HIV-1 IN protein is normally retained on integrated DNA and plays an active role in recruiting chromatin modifying enzymes and other host machinery to promote transcription at early times after DNA integration. That retention of IN, and the early activation of transcription, are significantly diminished in the QA mutant.

### Avian leukosis virus (ALV) has conserved lysine residues in CTD but is unaffected by conservative mutation

**Fig 7:**
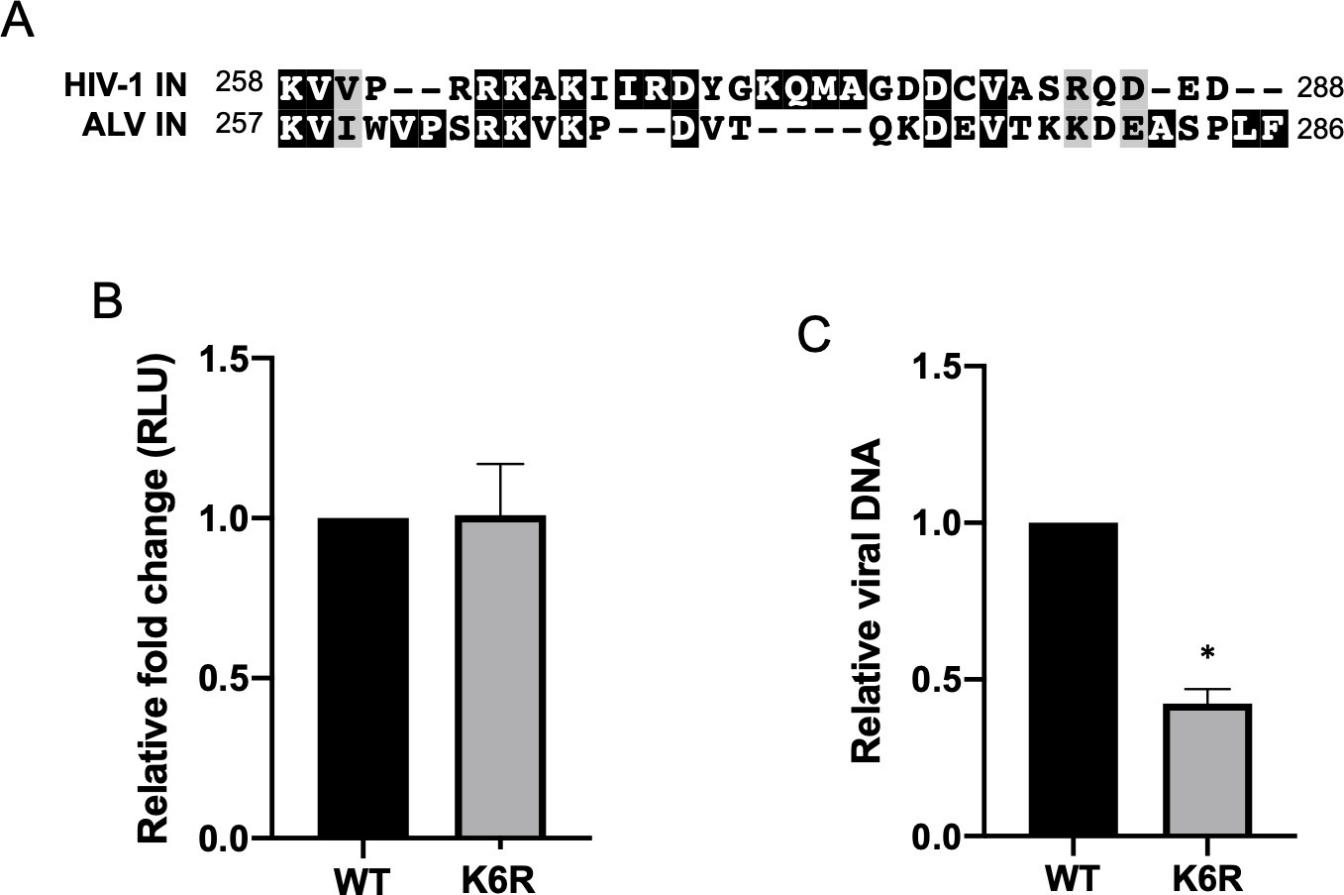
Avian leukosis virus (ALV) has conserved lysine residues in CTD but transduction is unaffected by conservative mutation of these residues. (A) Schematic highlighting conserved lysine residues in the C-terminal domain of HIV-1 and ALV IN. (B) The six lysine residues in the CTD of ALV IN were conservatively mutated to arginine in the pRIAS single-round infection vector carrying a luciferase reporter (K6R). VSV-G pseudotyped virus was produced and subsequently used to infect chick embryonic fibroblast (CEF) cells. The K6R mutation had no effect on successful infection as measured by luciferase reporter readout. (C) Total viral DNA present in cells infected with virus carrying either the WT or K6R mutant IN was measured by qPCR using primers against luciferase and normalized to GAPDH. Data shown are the average +/- SEs of four independent experiments run in duplicate. Statistical significance was gauged by paired t-test (* p < 0.01).

Amongst the various retroviral genera, the C-terminal domain (CTD) of the IN protein is the least conserved portion of the protein. Despite this, avian leukosis virus (ALV) has six lysines in the CTD, three of which are in sequences that can be aligned with sequences in the HIV-1 IN (K258, K264 and K266, Fig 7A). In addition, using available machine learning algorithms, two additional residues in the ALV IN CTD are predicted to be acetylated (K278, K279) [49]. It has not been experimentally established that these residues in ALV are acetylated. However, we wanted to determine if conservative mutation of these six lysine residues to arginine has an effect on successful transduction by ALV vectors, as seen here with HIV-1. To this end we generated an ALV single round infection vector carrying a luciferase reporter gene with these lysine residues conservatively mutated to arginine (K6R).

Virus was prepared by co-transfection of chick embryonic fibroblast (CEF) cells with ALV vectors encoding either the WT or K6R mutant IN with VSV-G envelope protein. At 2 days post-infection, luciferase reporter expression was assayed and was found to be indistinguishable between WT and mutant viruses (Fig 7B). Viral DNA levels in cells infected with the WT or K6R IN mutant virus were comparable, with a 2-fold decrease in mutant viral DNA levels on average (Fig 7C). We conclude that mutating these lysine residues of the ALV IN, unlike the HIV-1 IN, had a negligible effect on successful ALV expression. Thus, the effects of mutating lysine residues in the CTD of the HIV-1 IN are not seen for all retroviruses, but rather may be limited to select viruses. Other retroviral IN proteins may still modulate transcription but are seemingly not regulated by lysine acetylation.

## Discussion

We find that blocking acetylation of HIV-1 IN has very little effect on the catalytic activity of the IN protein *in vivo*. Our mutant vectors show at most a 2–3 fold decrease in integration frequency as compared to the WT control, which appears to be largely a consequence of decreased viral DNA levels available for integration (Fig 1B and 1C). This general phenotype was consistent across multiple cell types (S1 Fig). This finding is not in perfect agreement with a previous study that looked at the effect of IN acetylation using the same mutations. Terreni et. al. found a somewhat more significant decrease (approximately 5-fold) in integration efficiency that correlated well with their observed defect in reporter gene expression [42]. We suggest that the small difference in integration efficiency might be attributable to differences in virus preparation, viral titer, or corresponding multiplicity of infection. In any event, it is clear that the mutant IN protein in our hands is not drastically impaired for integration efficiency *in vivo*. The more profound difference in expression that we observe could be due to different reporter assays, or most likely, the timing of the readout of expression.

Various residues in the CTD of IN, including some of the residues mutated in this study, have been implicated as responsible for the phenotypes of class II IN mutants [7]. When these residues were mutated, IN binding to viral RNA was disturbed leading to the formation of non-infectious viral particles. We did not see this phenotype in our mutants. While we did not directly look at particle morphogenesis or viral RNA binding ability of our mutant IN proteins, we have shown that all mutants are competent for reverse transcription, nuclear import and integration implying that our mutant viruses do not fall into this class. We expect that this is very likely due to the fact that previous studies involved mutation of these lysine residues to alanine, while we have made more conservative lysine to arginine substitutions, retaining a basic residue at these sites. This allows us to block acetylation of these residues without dramatically impacting other properties of the CTD region of the protein.

Surprisingly, we found that the main impact of mutating known acetylated residues of HIV-1 IN was on post-integration proviral transcription (Fig 2). The mutation of the multiple acetylation sites of IN had a cumulative effect on transcription. Point mutation of individual lysine residues generally had a negligible effect on proviral transcription, except for the K258R mutation, while combined mutation of all known acetylated lysines caused a much larger defect. Based on labeling of various nascent transcripts produced from proviruses integrated by the quadruple acetylation (QA) mutant IN, we observed a substantial 80-fold average reduction in proviral transcription, even normalized to the amount of viral DNA available for transcription (Fig 2C). The acetylation deficient mutant IN generated proviruses that were profoundly defective for transcription. Interestingly, we observed that the effect of the QA mutation of IN on proviral gene expression is limited to early times. At later times after infection, viral expression from proviruses integrated by the QA mutant IN increased dramatically and reached nearly WT levels, while expression from WT viruses remains fairly constant over the 14-day time course (Fig 3A and 3B). This raises the possibility that integration, and consequently proviral transcription, may merely be delayed in the presence of the IN mutation. However, when we measure detectable proviral integrants over the same 14-day time course, we found that the level of proviral DNA is stable over time in both the WT and QA mutant viral infections with a consistent average 2-fold decrease in integration observed with the mutant virus (Fig 3C and 3D).

The effect of the IN mutations on early transcription was seen in a variety of cell lines of various morphologies and cell types. We saw similar phenotypes in all of the cell lines, though the magnitude of the effect did vary: it was exceedingly strong in Jurkat and HEK293T cells, strong in HeLa, and less dramatic in CEM cells (S1 Fig). The basis for the different strengths of effect on expression is not clear, but may reflect different levels of host transcription factors that are affected by IN.

There are a number of ways mutations in the HIV-1 IN protein might affect transcription. One possibility is that mutating the IN open reading frame in the virus might perturb the proper expression of the viral Tat protein. HIV-1 is a complex retrovirus, and the genome contains a series of overlapping ORFs. While the QA mutation does not directly alter the *tat* ORF, there is a region that regulates *tat* splicing that lies at the end of the *pol* ORF, where our various IN mutations are located [50]. Our mutations do not directly alter splice sites but do lie near a known splicing enhancer. To rule out confounding effects of our IN mutations on proper Tat expression, we measured mRNA levels of various viral transcripts to demonstrate that *tat* was not uniquely or more dramatically affected (Fig 2B and 2C). We further made use of a three-vector system in which the *gag-pol* genes are expressed from a separate plasmid from the transducing viral reporter genomes that are integrated. The reporter genes in the vectors are driven by distinctive promoters, either the CMV promoter or the human PGK promoter, which are not dependent on Tat protein levels. Further, one reporter genome used in these experiments was a so-called self-inactivating (SIN) vector and thus has no LTR promoter present after integration. In both cases, the proviral reporter gene was expressed significantly less efficiently when integrated by an acetylation-deficient mutant IN protein, indicating that the effect we see is independent of both Tat and the viral LTR promoter (Fig 4, S2 Fig).

Past studies have shown that HIV-1 integration is targeted to genomic locations by interaction of IN with host cell factors [28]. Thus, it is conceivable that ablating acetylation of the IN protein, or making any mutations in the IN sequence, might alter host factor binding affinity and thus integration site selection, and that our observed decrease in proviral transcription could be a result of position effects. However, we found that the distribution of proviral sites formed by the acetylation deficient QA mutant IN did not differ significantly from that of wild type, indicating that integration position effects are likely not the cause of decreased proviral transcription (Fig 5).

While high-throughput mapping of integration sites is informative for identifying potential differences in site selection, it only correlates integration sites with the locations of pre-infection histone modifications. To determine the chromatin status of the proviral DNA itself in QA and WT virus, we performed ChIP with antibodies specific for an active histone modification, H3K27ac. We demonstrate that the decrease in proviral transcription we observed in the acetylation deficient IN mutant virus is accompanied by a decrease in H3K27ac deposited on the viral LTR at early times post-infection. These modifications equilibrated to wild type levels as transcription of mutant proviruses increased at later times post-infection (Fig 6A).

The concordant changes in transcription and histone modifications on the proviruses integrated by an acetylation-deficient mutant observed here raise the possibility that IN is altering proviral transcription by directly influencing histone modifications in the viral LTR promoter. One plausible hypothesis is that the introduced mutations disrupt binding of specific host factors. The most well-known binding partner of IN is LEDGF, which is thought to target integration site selection to active chromatin via a tethering mechanism [51]. Another binding partner is INI-1 (hSNF5), a subunit of the Snf/Swi chromatin remodeling complex [52]. Wild type HIV-1 IN has also been reported to interact with numerous other chromatin-modifying enzymes such as histone acetyltransferases and histone deacetylases [53–55]. These or other transcription factors may be recruited by WT IN to promote early activation of transcription from the newly-integrated proviruses.

Extensive evidence implicates post-translational modifications, such as acetylation, in mediating protein-protein interactions. Blocking acetylation of IN may cause the mutant protein to bind different host factors or to lose binding affinity to factors that the WT IN protein would typically bind. A previous yeast two-hybrid screen, comparing host factors interacting with wild type IN protein and a constitutively acetylated IN, identified the universal transcriptional corepressor KAP1 as a binding partner of acetylated IN [56]. KAP1 was shown to negatively regulate HIV-1 replication through recruitment of the histone deacetylase HDAC1, which could deacetylate IN as a negative feedback [57,58]. These binding proteins would presumably promote silencing of the provirus, rather than promote early expression that we observed here. To preliminarily investigate any differential host factor interactions induced by the QA mutation in IN, we performed a co-immunoprecipitation of WT and QA mutant IN followed by mass-spectrometry to identify binding partners. However, both WT and mutant IN proteins appeared to bind largely the same host factors, including LEDGF. We could not detect bound KAP1 or HDAC1 in these experiments.

An alternative mechanism is that the acetylation deficient mutant QA IN could be binding host cell factors equally as well as the WT protein, but have decreased binding affinity for viral DNA. Mutations in the CTD of HIV-1 IN are known to participate in IN-DNA interactions and the same mutations described here that block acetylation have also been shown to reduce viral DNA binding affinity *in vitro* [41]. In agreement with these experiments, we find that our QA mutation in IN does lead to a significant reduction of IN bound to the viral LTR *in vivo* based on ChIP experiments at 24 hours post-infection (Fig 6B, S3 Fig). The nature of the binding of IN to the viral DNA in this setting is uncertain. The IN protein at this time point is likely bound to both unintegrated and integrated DNA, and the QA mutation may affect the binding to both forms. We tested for the binding of IN exclusively to unintegrated DNA by using the integrase inhibitor raltegravir, or the catalytically dead D64A mutant, and here too we observed much less binding by the QA mutant compared with WT. However, the majority of the viral DNA at this time point is integrated, suggesting that the QA mutation is largely affecting the retention of the IN on the proviral DNA. Indeed, when integration is blocked, the amount of DNA bound by either WT or QA mutant IN is less than when integration is allowed, indicating that most of the ChIP signal is coming from integrated proviral DNA.

We would expect that the affinity of integrase for unintegrated linear DNA could very well be different from the affinity of integrase for the DNA of the recently-inserted provirus. The structure of these two viral DNA forms are very different. The integrase in the context of the unintegrated PIC is in an oligomeric form bound to the termini of the linear DNA. After integration, we do not know where the integrase is bound on the provirus, nor what part of the integrase protein is involved in that binding. The integrase could be bound to the cruciform at the base of a viral DNA loop; or bound to the residual terminal DNA sequences of the provirus. It might well be in a different oligomeric state. It may be binding the gapped DNA left at the junctions formed by the strand transfer reaction prior to repair of the provirus, and would be ejected post-repair, limiting its effects on transcription to early times post-integration. Whatever the DNA structure bound, the QA mutation seems to act to reduce the lifetime of the IN on the viral DNA.

Almost immediately upon formation by reverse transcription and entry into the nucleus, viral DNA is loaded with histones and efficiently silenced through the presence of repressive histone modifications such as H3K9me3 [10,11]. This silencing is alleviated upon integration of the viral DNA into the host genome. How the process of integration into host DNA activates transcription is unknown. It is possible that the IN protein is normally retained and plays a direct role in reversing the suppressive marks and promoting the formation of activation marks on the viral histones.

While we initially mutated these four lysine residues in the CTD of HIV-1 IN to look at the effect of blocking acetylation, it is an important caveat that our work does not preclude that these mutations may be causing a phenotype unrelated to the presence of acetylation. Residues in the CTD of HIV-1 IN are known to be important for IN-IN interactions within the intasome and our mutations could be perturbing these interactions. However, we believe that the comparable efficiency of integration of our wild type and mutant suggests that there are no large-scale perturbations to the intasome. Residues in the CTD of HIV-1 IN have also been implicated in post-integration repair through recruitment of Ku70 [59]. It plausible that a defect in gap repair could affect subsequent viral gene expression. Our preliminary co-IP studies show that Ku70 binds both WT and QA mutant IN suggesting that our introduced mutations are not perturbing this recruitment. However, it remains possible that post-integration repair may be otherwise affected by our introduced mutations and the reduction in levels of bound IN. Lastly, acetylation is not the only PTM that targets lysines and it is possible that we could be ablating an unknown PTM on one of these residues that mediates this phenotype. There may be many more consequences of the retention of IN on the provirus than those we have detected here.

In summary, our work here suggests that HIV-1 IN may play a more active role in establishing a permissive chromatin environment for transcription than previously thought and that PTMs of IN, such as acetylation, may coordinate this function. We suspect the regulation of this function of IN by acetylation is specific to HIV-1. We found that despite conservation of several lysine residues in the CTD of the ALV IN protein, mutation of these residues has no effect on any stage of the viral life cycle including early viral transcription (Fig 7). Further, it has previously been shown that a lysine residue in MLV IN, homologous to K266 in HIV-1 IN, is similarly acetylated but blocking this acetylation via mutation had little effect on viral gene expression [60]. It remains possible, given the conserved functions of the IN protein across retroviral genera, that other IN proteins also regulate proviral transcription, but it seems that these conserved residues or their post-translational modifications are not mediating this function.

We suggest that when we introduce mutations that block known acetylation of the HIV-1 IN protein, IN is no longer retained on the proviral DNA and is unable to recruit host factors that would normally play a role in activating proviral transcription through chromatin modification. Thus, the repressive chromatin state present on the unintegrated viral DNA may remain intact for longer times, causing low proviral transcription even after integration. Because the provirus is most often integrated in active chromatin regions, mutant proviruses will eventually become active through spreading of the adjacent host chromatin environment, resulting in the observed gradual return in proviral transcription at later times post-integration. The findings here define yet another function for the IN protein, the acceleration of viral gene expression immediately after its insertion of the viral DNA into the host genome.

## Methods and materials

### Cell lines

HEK293T and HeLa cells were cultured in DMEM media supplemented with 10% FBS and 1% pen-strep at 37°C and 5% CO_2_. Jurkat and CEM cells were cultured in RPMI 1640 supplemented with 10% FBS at 37°C and 5% CO_2._ Chick embryo fibroblasts were cultured in Media 199 supplemented with 1% calf serum and 1% chick serum at 39°C.

### Viral plasmids

The pNL4.3.Luc.R-E- vector was obtained from the NIH AIDS Reagent Program (#3148). The replication-defective pNL4.3 luciferase reporter construct lacks envelope and expresses firefly luciferase from the *nef* open reading frame.

The packaging construct pCMV delta R8.91 expressing HIV-1 *gag-pol* was used to generate virus preparations with various reporter constructs. Two different reporter constructs were used to separate effects of Tat dependency and the LTR promoter on proviral transcription. The pLVX-IRES-ZsGreen plasmid is a minimal lentiviral expression vector consisting of HIV-1 packaging and LTR sequences and an internal CMV-promoter driven ZsGreen reporter gene (Takara Bio). Second, a self inactivating (SIN) reporter construct, pRRLSIN.cPPT.PGK-GFP.WPRE (Addgene 12252), was used to deliver a GFP reporter gene driven by a PGK promoter.

ALV viral constructs were based on the single-round infection vector pRIAS which lacks envelope and contains a luciferase reporter gene [61,62].

Conservative point mutations in the integrase coding sequence were introduced into all plasmids using PCR site-directed mutagenesis (See S3 Table for primer sequences used). Combinedmutations of known acetylated lysine residues were generated using a custom gBlock (IDT) spanning the region.

### Transfection, virus preparation and infection

All DNA transfections were performed using Lipofectamine 3000 (Life Technologies) following the manufacturer’s protocol.

To generate pseudotyped HIV-1 virus for infections, the viral vector pNL4.3.Luc.R-E- (or derivative mutant vector) was co-transfected with a plasmid encoding VSV-G (pMD2.G) in HEK293T cells. To package lentiviral reporters, pCMVdeltaR8.91 encoding HIV-1 *gag-pol* with WT or mutant IN sequence was co-transfected with the appropriate reporter construct–either pLVX-IRES-ZsGreen or pRRLSIN.cPPT.PGK-GFP.WPRE–and plasmid pMD2.G expressing VSV-G envelope. Pseudotyped ALV was produced in chick embryonic fibroblast (CEF) cells by co-transfection of the pRIAS viral vector and pMD2.G.

Viral supernatants were collected 48 hours post-transfection and treated with RQ1 DNase (Promega) to remove residual plasmid DNA. Viruses were used immediately after collection and diluted 3-fold with cell culture medium for infections. Virus quantities were normalized prior to infection by quantification of viral RNA genome content by qRT-PCR.

Successful viral transduction was assayed using either a luciferase assay or flow cytometry depending on the reporter construct used. Luciferase activity was assayed using the Promega Luciferase Assay System (Cat# E4550). ZsGreen or GFP positive cells and mean fluorescence intensity (MFI) were measured by flow cytometry using an automated cell analyzer (LSRII, BD Bioscience).

### Quantitative PCR for viral intermediate analysis

To assay viral DNAs formed after acute infections, total cell DNA was collected from infected cells using the Qiagen DNeasy Blood and Tissue kit. Subsequent quantitative PCR was performed on genomic DNA using FastStart Universal SYBR Green Mastermix (Bio-Rad) according to manufacturer’s protocol on ABI 7500 Fast Real Time PCR System. Total viral DNA was quantified using primers complementary to the luciferase reporter sequence. Late reverse transcription products were assayed with primers in the LTR. 2-LTR circles were quantified as previously published and normalized to a housekeeping gene [63]. Integrated provirus was measured using the Alu-gag nested PCR method [44,64]. See S4 Table for primer sequences used for all assays. All qPCR assays for viral DNA intermediates at two days post-infection were quantified using the 2^-ΔΔCt^ method and the results were first normalized for total DNA input by a host housekeeping gene and then expressed as fold change relative to the WT control.

To monitor integration timing dynamics, DNA was collected from infected HeLa cells at 2, 4, 7, 10 and 14 days post-infection. Integration frequency was measured by Alu-gag nested PCR. The 2^-ΔΔCt^ method for quantification was used to score levels of integrated DNA, and expressed relative to the last collected time point as a measure of the final levels produced by the single-round vectors. Finally, as a more direct comparison, the ratio of integration quantified in cells infected with the mutant QA IN virus relative to WT virus was calculated.

A minimum of three biological replicates were performed for each assay. Biological replicates consist of completely independent virus preparations and cells, infections, and assays. DNA preparations and qPCR assays were done independently. Within each biological replicate there are technical duplicates for consistency. A single factor ANOVA analysis was used to confirm significant changes within each experiment (p value < 0.01). When appropriate, this analysis was followed by pairwise comparisons using a two-tailed t-test assuming unequal variance in Prism.

### Reverse transcription-qPCR and EU labeling of nascent mRNA

Steady state RNA levels were measured by collecting total RNA from infected cells at designated times post-infection using a standard Trizol protocol. Reverse transcription was subsequently performed using random hexamer primers and Maxima H Reverse Transcriptase (Thermo Fisher).

5-ethynyl uridine (EU) labeling was performed using the Click-It Nascent RNA Capture Kit according to manufacturer’s protocol (Thermo Fisher). Briefly, two days post-infection, cells were pulse-labeled by incubation for 4 hours in medium containing 0.5 mM EU ribonucleotide homologs containing a reactive alkyne group. Total RNA was collected, biotin azide was attached to the incorporated EU ribonucleotides and the RNA was subsequently recovered by binding with and elution from streptavidin beads. Viral RNA was then quantified by qRT-PCR.

Quantitative RT-PCR was performed after reverse transcription of RNA preparations to produce either total cDNA or cDNA generated from streptavidin pulldown from EU-labeled RNA. The subsequent PCR used various primers against viral transcripts allowing for detection of spliced *tat* mRNA, luciferase mRNA, or envelope mRNA as well as *gag* mRNA and all LTR containing sequences (S4 Table).

To monitor viral transcript levels, RNAs were collected at two days post-infection and quantified by qRT-PCR assays using the 2^-ΔΔCt^ method to first normalize for total cDNA input and then to calculate fold change relative to the WT condition. Viral mRNA levels were further normalized to total proviral DNA as measured by qPCR against luciferase DNA to standardize to the levels of viral DNA available for transcription.

To analyze proviral transcription dynamics over time, mRNA was collected at 2, 4, 7, 10 and 14 days post-infection and subsequently quantified by qRT-PCR using primers specific for the spliced *tat* message. Viral mRNA levels were quantified for WT and QA mutant IN viruses individually over the time course using the 2^-ΔΔCt^ method and normalized to viral mRNA levels present at the final time point. For direct comparison, the mRNA levels produced by the QA mutant IN were calculated as a ratio relative to WT at each individual time point.

A minimum of three biological replicates was performed from independently infected cells. RNA preparations, cDNA preparations and qPCR assays were done independently. Within each qPCR assay technical duplicates were also carried out to confirm consistency. A single factor ANOVA analysis was used to confirm significant changes within each experiment (p value < 0.01). When appropriate, this analysis was followed by pairwise comparisons using a two-tailed t-test assuming unequal variance in Prism.

### Integration site next-generation sequencing (NGS) library preparation

Sequencing libraries of viral-host DNA junctions were prepared as described previously [65,66]. Specifically, five micrograms of purified genomic DNA from infected or control HeLa cells was randomly sheared using Branson 450 Digital sonifier. Sheared ends of DNA were subsequently repaired and A-tailed with Klenow polymerase fragment exonuclease, and custom oligonucleotide adaptors were ligated onto DNA ends. Nested PCR was performed to enrich the library for proviral-host genome junctions. The first round of PCR reactions made use of a viral-specific primer complementary to a sequence near the 3’ end of the HIV-1 LTR and an adaptor primer, and consisted of 20 cycles of amplification. Second round PCR consisted of 20 cycles using one primer nearer to the 3’end of the LTR, paired with a barcoded adaptor-specific primer, such that the products would include the adaptor barcode sequence as well as 40 nucleotides of the viral LTR. See S5 Table for library adaptor and primer sequences. Deep sequencing was performed using the Illumina MiSeq platform. Three unique NGS libraries were prepared from independently infected cells and sequenced in distinct MiSeq runs.

### Integration site mapping data analysis

Viral-host DNA junction reads were demultiplexed by unique dual barcodes and filtered to exclude reads not containing an initial viral LTR sequence at the host junction using a custom python script [67]. Reads from filtered FASTA files were then trimmed to remove adaptor and viral sequences. Reads of less than 20 nucleotides after adaptor removal and trimming were then removed. Remaining reads were mapped to the hg38 human genome using Bowtie2 [68,69].

Using Bowtie2, sequences were aligned end-to-end using a seed length of 28 nucleotides, with a maximum of 2 mismatches permitted in the seed. Alignments for reads were suppressed if more than one reportable alignment existed to prevent multiple mapping and ensure that reads corresponded to unique integration sites. Multi-mapping reads were written to a separate FASTA file and subsequently mapped to the RepeatMasker genome to analyze integrations into repeat sequences [70].

### Analysis of integration sites with respect to genomic annotations

Genomic coordinates of RefSeq genes, transcription start sites, DNase hypersensitivity regions and CpG islands were extracted from the hg38 genome assembly via the UCSC Genome Browser. Genomic locations of RNA polymerase II binding and histone modifications was extracted from ENCODE data sets (Pol II: ENCFF246QVY; H3K27Ac: ENCFF113QJM; H3K9me3: ENCFF712ATO; H3K36me3: ENCFF864ZXP; H3K4me3: ENCFF862LUQ). Genomic coordinates of “super enhancers” in HeLa cells were extracted from the SEA 3.0 database [71]. Distance of integration to nearest feature was calculated using BedTools [72]. A matched random control (MRC) data set of comparable size was generated with BedTools Random command.

To gauge the statistical significance of differences in integration patterns between WT IN and acetylation deficient (QA) IN we used a paired t-test of three independent replicate data sets for each condition (S2 Table). A one-sample t-test was used to compare integration distribution between experimental samples and MRC.

### Chromatin immunoprecipitation and qPCR

Infected HeLa cells were crosslinked using 1% formaldehyde for 10 minutes at 37°C. Cells were subsequently lysed with ChIP lysis buffer (50 mM Tris-HCl pH 8.0, 1% SDS, 10 mM EDTA and protease inhibitor cocktail). Chromatin was sheared by sonication with a Branson 450 Digital Sonifier to an average size of 500–1000 nucleotides in length. 25 ug of chromatin as measured by spectrometer was mixed with 2 μg of various specific antibodies and incubated overnight at 4°C. For immunoprecipitation of H3K27ac, a ChIP-validated polyclonal mouse antibody was used (EMD Millipore). Integrase IP was performed with either a mouse monoclonal antibody (IN-2; abcam ab66645) or polyclonal antibodies (IN-9379). ChIP with histone H3 (abcam ab10799) and control mouse IgG (abcam 37355) antibodies were performed in parallel as a positive and negative control respectively. Post-incubation, the chromatin-antibody mixture was mixed with Protein A and Protein G beads and incubated with rotation for 2 hours at 4°C. Beads were then washed extensively as described [11]. Crosslinks were reversed overnight by incubation with Proteinase K and RNase A. DNA was eluted and purified using the Qiagen PCR Purification Kit. Immunoprecipitated viral DNA was then quantified via qPCR using LTR-specific primers and was calculated as a percent relative to input viral DNA. A minimum of two independent biological replicates were performed in duplicate.

The monoclonal antibody used for HIV-1 IN immunoprecipitation was validated for comparable binding to WT and acetylation deficient QA mutant IN by Western blot. The WT or mutant IN open reading frame was cloned into a pJET mammalian expression vector and HA-tagged at the C-terminal end. Equal quantities of plasmid were transfected into HEK293T cells. Transfected cells were lysed and used for Western blot with antibodies targeting the HA-tag or IN.

## Supporting information

S1 Fig Introduction of QA mutation in HIV-1 IN causes similar phenotype of defective transcription across multiple cell lines. Viruses produced from pNL4.3R-E- viral vector with either a WT IN or QA mutant IN sequence were used to infect either (A) HeLa, (B) HEK293T, (C) CEM or (D) Jurkat cells. All infected cells were collected at two days post-infection. From each independent experiment, cells were collected to measure luciferase activity, and genomic DNA and total RNA was isolated in parallel. From genomic DNA, total reverse transcription (RT) products as well as integrated provirus were quantified by qPCR. Transcription was roughly quantified by measurement of steady state *tat* mRNA levels by RT-qPCR. All measurements were done in parallel. Data shown is average of a minimum of three independent biological replicates +/- SEs. Statistical significance was gauged by paired t-test.
(TIF)

S2 Fig The effect of the QA mutation in IN on proviral transcription is independent of LTR promoter. The QA mutation in the CTD of IN was introduced into pCMV-delta-R8.9 vector expressing only HIV-1 *gag-pol*. VSV-G pseudotyped virions were produced by co-transfecting the plasmid expressing either WT or QA mutant *gag-pol* along with a minimal self-inactivating (SIN) viral construct carrying a GFP reporter gene driven by a human PGK promoter. Infected HeLa cells were collected two days post-infection. (A) Representative flow cytometry data of one independent experiment is shown. (B) Average percent of GFP positive cells and (C) mean fluorescence intensity (MFI) after infection with viruses carrying WT or QA mutant IN. Data shown is average +/- SEs of three independent experiments run in duplicate.
(TIF)

S3 Fig Less proviral DNA integrated by QA mutant IN retains bound IN protein than DNAs integrated by WT IN at 24 hours post-infection. HeLa cells infected with virus expressing either a WT or QA mutant IN were collected at 24 hours post-infection. Quantity of viral DNA bound to IN protein was estimated via chromatin immunoprecipitation (ChIP) using a polyclonal antibody against the IN protein followed by qPCR using LTR-specific primers. Data shown is average of two independent biological replicate experiments run in duplicate +/- SEs.
(TIF)

S1 Table Total number of unique integrations sequenced, summed from three independent biological replicates. (DOCX)

S2 Table Statistical analysis of integration frequency near common genomic features for viruses carrying WT or QA acetylation-deficient mutant IN. Paired two-tailed t-test of three independent biological replicate NGS experiments.
(DOCX)

S3 Table Primers using for PCR site-directed mutagenesis to generate point mutations in HIV-1 IN sequence. Mutated base is highlighted in red.
(DOCX)

S4 Table Primer sequences used for qPCR analysis of viral DNA intermediates and transcripts. (DOCX)

S5 Table Adaptor and primer sequences used for construction of integration site mapping NGS libraries. (DOCX)
