# Supplementary figures and images for: Mutations altering acetylated residues in the CTD of HIV-1 integrase cause defects in proviral transcription at early times after integration of viral DNA
Source: PLoS Pathog. 2020 Dec 22;16(12):e1009147. doi: 10.1371/journal.ppat.1009147 (PMC7787678; doi:10.1371/journal.ppat.1009147)

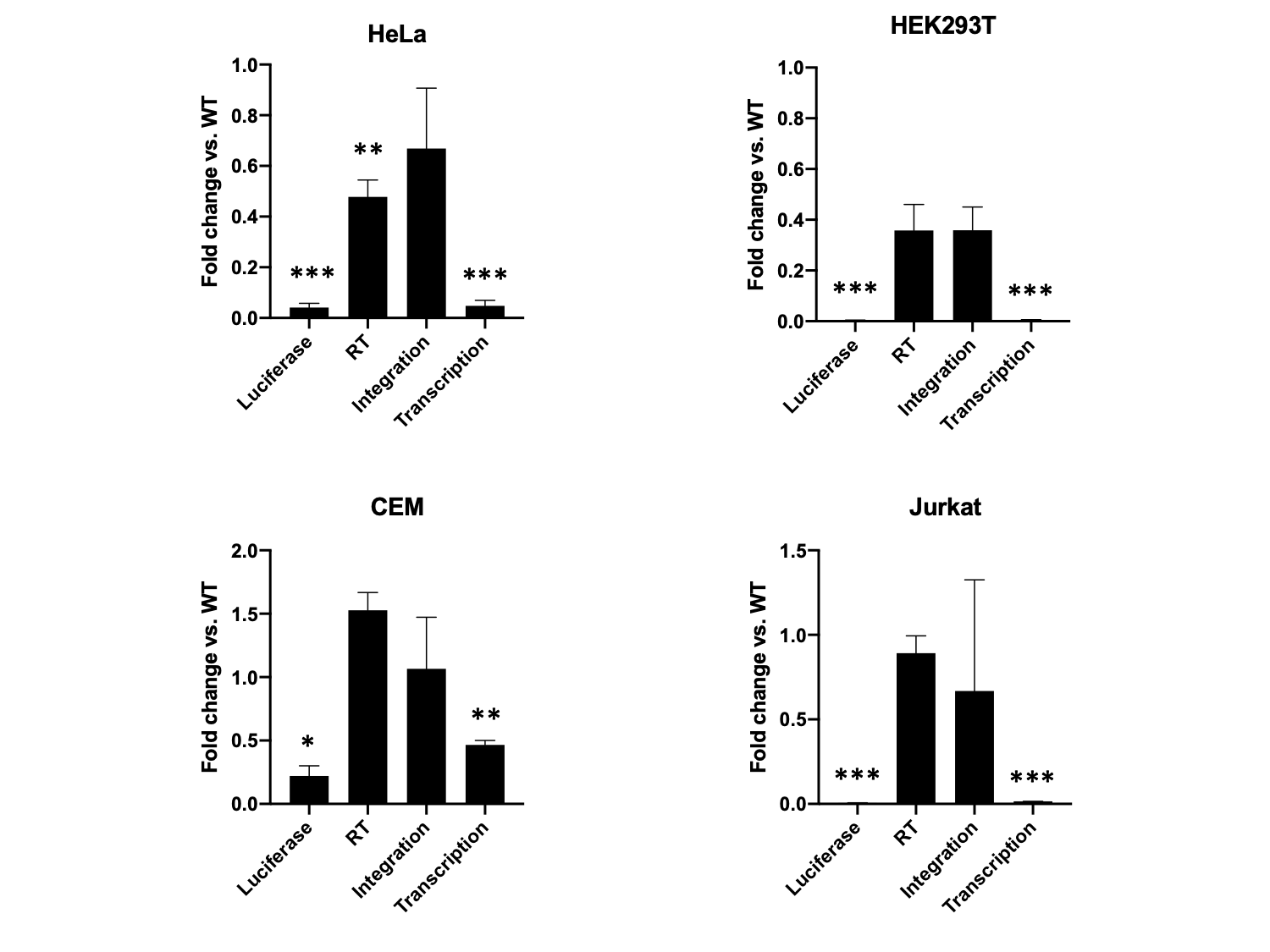

Supplement: S1 Fig — Viruses produced from pNL4.3R-E- viral vector with either a WT IN or QA mutant IN sequence were used to infect either (A) HeLa, (B) HEK293T, (C) CEM or (D) Jurkat cells. All infected cells were collected at two days post-infection. From each independent experiment, cells were collected to measure luciferase activity, and genomic DNA and total RNA was isolated in parallel. From genomic DNA, total reverse transcription (RT) products as well as integrated provirus were quantified by qPCR. Transcription was roughly quantified by measurement of steady state tat mRNA levels by RT-qPCR. All measurements were done in parallel. Data shown is average of a minimum of three independent biological replicates +/- SEs. Statistical significance was gauged by paired t-test. (TIF) [file ppat.1009147.s001.tif]

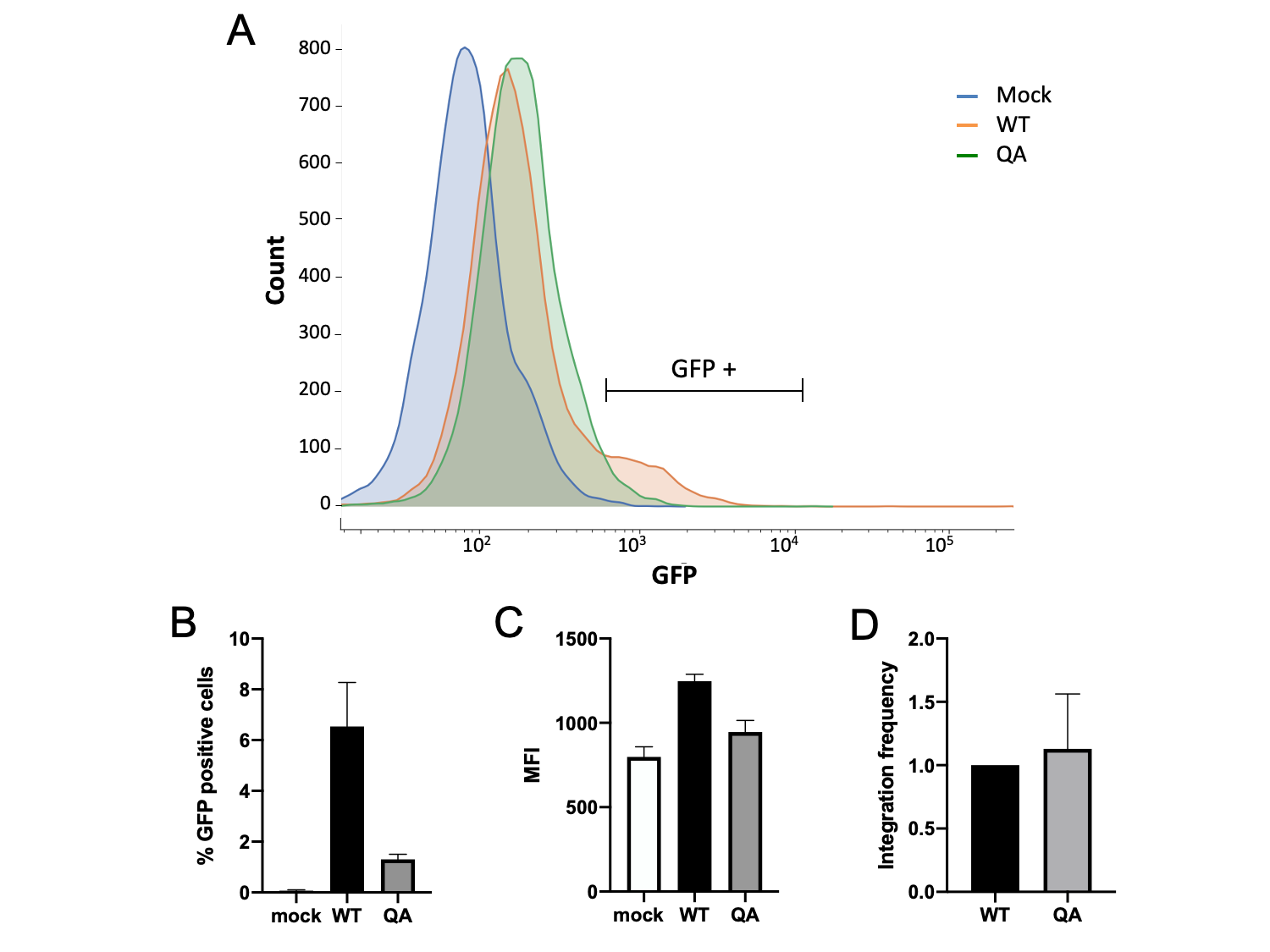

Supplement: S2 Fig — The QA mutation in the CTD of IN was introduced into pCMV-delta-R8.9 vector expressing only HIV-1 gag-pol. VSV-G pseudotyped virions were produced by co-transfecting the plasmid expressing either WT or QA mutant gag-pol along with a minimal self-inactivating (SIN) viral construct carrying a GFP reporter gene driven by a human PGK promoter. Infected HeLa cells were collected two days post-infection. (A) Representative flow cytometry data of one independent experiment is shown. (B) Average percent of GFP positive cells and (C) mean fluorescence intensity (MFI) after infection with viruses carrying WT or QA mutant IN. Data shown is average +/- SEs of three independent experiments run in duplicate. (TIF) [file ppat.1009147.s002.tif]

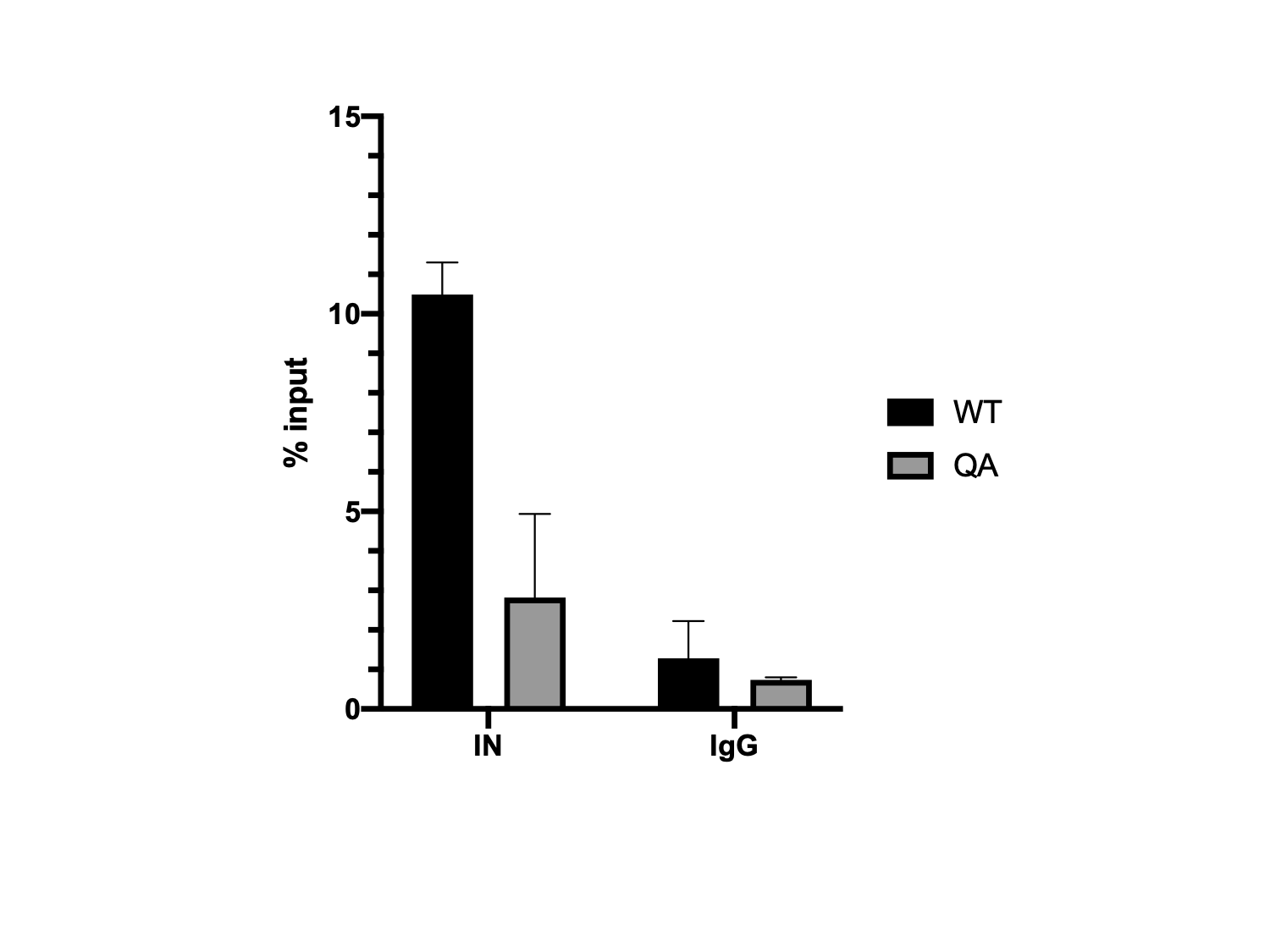

Supplement: S3 Fig — HeLa cells infected with virus expressing either a WT or QA mutant IN were collected at 24 hours post-infection. Quantity of viral DNA bound to IN protein was estimated via chromatin immunoprecipitation (ChIP) using a polyclonal antibody against the IN protein followed by qPCR using LTR-specific primers. Data shown is average of two independent biological replicate experiments run in duplicate +/- SEs. (TIF) [file ppat.1009147.s003.tif]
