# Supplementary material for: Mutations altering acetylated residues in the CTD of HIV-1 integrase cause defects in proviral transcription at early times after integration of viral DNA
Source: PLoS Pathog. 2020 Dec 22;16(12):e1009147. doi: 10.1371/journal.ppat.1009147 (PMC7787678; doi:10.1371/journal.ppat.1009147)
Supplement: S1 Table — (DOCX) [file ppat.1009147.s004.docx]

**S1 Table**: Total number of unique integrations sequenced, summed from three independent biological replicates.

|  | **WT** | **QA** | **MRC** |
| --- | --- | --- | --- |
| Total unique integrations | 9507 | 16565 | 10000 |
| RefSeq genes | 6890 | 10351 | 5271 |
| DNase HS (1kb) | 1132 | 2386 | 1238 |
| TSS (1kb) | 197 | 445 |  |
| TSS (5kb) | 1250 | 2052 |  |
| CpG islands (1kb) | 270 | 576 | 217 |
| CpG islands (5kb) | 1730 | 2243 | 760 |
| RNA polymerase II (1kb) | 267 | 555 | 295 |
| H3K27ac (1kb) | 1013 | 1607 | 527 |
| H3K36me3 (1kb) | 1320 | 1338 | 326 |
| H3K4me3 (1kb) | 501 | 1012 | 432 |
| H3K9me3 (1kb) | 66 | 94 | 102 |
| Super enhancers | 615 | 685 | 127 |
