# Supplementary material for: Mutations altering acetylated residues in the CTD of HIV-1 integrase cause defects in proviral transcription at early times after integration of viral DNA
Source: PLoS Pathog. 2020 Dec 22;16(12):e1009147. doi: 10.1371/journal.ppat.1009147 (PMC7787678; doi:10.1371/journal.ppat.1009147)
Supplement: S2 Table — Paired two-tailed t-test of three independent biological replicate NGS experiments. (DOCX) [file ppat.1009147.s005.docx]

**S2 Table**: Statistical analysis of integration frequency near common genomic features for viruses carrying WT or QA acetylation-deficient mutant IN. Paired two-tailed t-test of three independent biological replicate NGS experiments.

|  | WT vs. MRC | QA vs. MRC | WT vs. QA |
| --- | --- | --- | --- |
| RefSeq genes | 0.034 | 0.058 | 0.5858 |
| DNase HS (1kb) | 0.7431 | 0.9571 | 0.826 |
| TSS (1kb) | 0.02236 | 0.0181 | 0.3054 |
| TSS (5kb) | 0.0339 | 0.0766 | 0.3737 |
| CpG islands (1kb) | 0.2293 | 0.6058 | 0.8831 |
| CpG islands (5kb) | 0.0872 | 0.1384 | 0.2878 |
| RNA polymerase II (1kb) | 0.2832 | 0.6232 | 0.0429 |
| H3K27ac (1kb) | 0.0549 | 0.1759 | 0.4692 |
| H3K36me3 (1kb) | 0.0211 | 0.1432 | 0.2146 |
| H3K4me3 (1kb) | 0.2865 | 0.535 | 0.6461 |
| H3K9me3 (1kb) | 0.0784 | 0.0372 | 0.6093 |
| Super enhancers | 0.0731 | 0.1892 | 0.3422 |
