# Supplementary material for: Mutations altering acetylated residues in the CTD of HIV-1 integrase cause defects in proviral transcription at early times after integration of viral DNA
Source: PLoS Pathog. 2020 Dec 22;16(12):e1009147. doi: 10.1371/journal.ppat.1009147 (PMC7787678; doi:10.1371/journal.ppat.1009147)
Supplement: S3 Table — Mutated base is highlighted in red. (DOCX) [file ppat.1009147.s006.docx]

**S3 Table**: Primers using for PCR site-directed mutagenesis to generate point mutations in HIV-1 IN sequence. Mutated base is highlighted in red.

| **Mutation** | **Primer sequence (5’-3’)** |
| --- | --- |
| K258R | GATAATAGTGACATAAGAGTAGTGCCAAGAAG |
|  | CTTCTTGGCACTACTCTTATGTCACTATTATC |
| K264R | GTAGTGCCAAGAAGAAGAGCAAAGATCATCAGGG |
|  | CCCTGATGATCTTTGCTCTTCTTCTTGGCACTAC |
| K266R | GCCAAGAAGAAAAGCAAGGATCATCAGGGATTATGG |
|  | CCATAATCCCTGATGATCCTTGCTTTTCTTCTTGGC |
| K273R | CAGGGATTATGGAAGACAGATGGCAGGTG |
|  | CACCTGCCATCTGTCTTCCATAATCCCTG |
