# Supplementary material for: Mutations altering acetylated residues in the CTD of HIV-1 integrase cause defects in proviral transcription at early times after integration of viral DNA
Source: PLoS Pathog. 2020 Dec 22;16(12):e1009147. doi: 10.1371/journal.ppat.1009147 (PMC7787678; doi:10.1371/journal.ppat.1009147)
Supplement: S4 Table — (DOCX) [file ppat.1009147.s007.docx]

**S4 Table**: Primer sequences used for qPCR analysis of viral DNA intermediates and transcripts.

| **Target** | **Primer sequence (5’-3’)** |
| --- | --- |
| Late RT | TGTGTGCCCGTCTGTTGTGT |
|  | GAGTCCTGCGTCGAGAGATC |
| Luciferase | CGTCTTTCCGTGCTCCAAAAC |
|  | CAAAGGATATCAGGTGGCCC |
| 2LTR circles | AACTAGGGAACCCACTGCTTAAG |
|  | TCCACAGATCAAGGATATCTTGTC |
| Alu-gag nest 1 | GCCTCCCAAAGTGCTGGGATTACAG |
|  | GCTCTCGCACCCATCTCTCTCC |
| Alu-gag nest 2 | GCCTCAATAAAGCTTGCCTTGA |
|  | TCCACACTGACTAAAAGGGTCTGA |
| *Tat* mRNA | GTTTGTTTCATGACAAAAGCCTTA |
|  | CTATTCCTTCGGGCCTGTC |
| *Gag* mRNA | GAATTTGCCAGGAAGATGGA |
|  | GCAGCCAATGTGAGTCAACA |
| *Env* mRNA | TGCTGAAGCGCGCACGGCAAG |
|  | GCTACTACTAATGCTACTATTGC |
