# Supplementary material for: Mutations altering acetylated residues in the CTD of HIV-1 integrase cause defects in proviral transcription at early times after integration of viral DNA
Source: PLoS Pathog. 2020 Dec 22;16(12):e1009147. doi: 10.1371/journal.ppat.1009147 (PMC7787678; doi:10.1371/journal.ppat.1009147)
Supplement: S5 Table — (DOCX) [file ppat.1009147.s008.docx]

**S5 Table**: Adaptor and primer sequences used for construction of integration site mapping NGS libraries.

| **Primer name** | **Primer sequence** |
| --- | --- |
| Adaptor short arm | P-GATCGGAAGAGCAAAAAAAAAAAAAAAA |
| Adaptor long arm | CAAGCAGAAGACGGCATACGAGATnnnnnnGTGACTGGAGTTCAGACGTGTGCTCTTCCGATC*T |
| PCR-1-F | TGTGACTCTGGTAACTAGAGATCCCTC |
| PCR-1-R | CAAGCAGAAGACGGCATACGAGAT |
| PCR-2-F | AATGATACGGCGACCACCGAGATCTACACTCTTTCCCTACACGACGCTCTTCCGATCTGAGATCCCTCAGACCCTTTTAGTCAG |
| PCR-2-R | CAAGCAGAAGACGGCATACGAGATnnnnnn |

nnnnnn denotes a 6-bp unique barcode, P denotes phosphorylation and * denotes a phosphorothioate bond
